# Supplementary material for: Efficient cross-trait penalized regression increases prediction accuracy in large cohorts using secondary phenotypes
Source: Nat Commun. 2019 Feb 4;10:569. doi: 10.1038/s41467-019-08535-0 (PMC6361917; doi:10.1038/s41467-019-08535-0)
Supplement: Supplementary file 1 — Supplementary Information [file 41467_2019_8535_MOESM1_ESM.pdf]

Supplementary Information

Efficient Cross-Trait Penalized Regression Increases Prediction Accuracy in Large Cohorts using Secondary Phenotypes

Wonil Chung et al.

Supplementary Figure 1. Effects of number of causal SNPs (per-SNP heritability) on prediction accuracy. We set 2 traits, 14,800 training samples, 5,000 SNPs, 50% heritability.

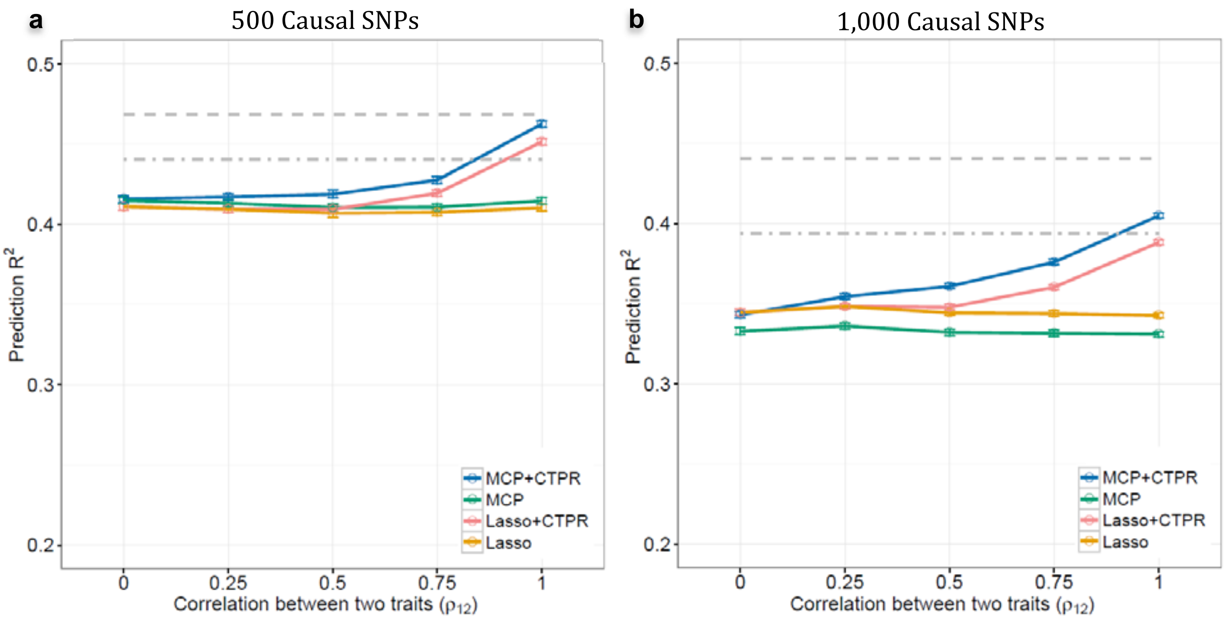

For more detailed evaluation of our methods, we used individual-level genotype data for all traits with a smaller number of SNPs and repeated 100 times for each scenario. We sampled 14,800 individuals from the NHS/HPFS/PHS cohort data for a training set, 5,900 individuals for a validation set with randomly selected 5,000 SNPs that are all common and in low LD. We simulated two phenotypes with 500 causal SNPs. All error bars represent standard errors of prediction R^2.

(a) We first considered the scenarios with 14,800 training samples (7,400 for the primary trait and the other 7,400 for the secondary trait), 5,000 SNPs and 500 causal SNPs. We set trait-heritability for both traits to 50% and ranged the genetic correlation between two traits from 0 to 1. As mentioned in the manuscript, increase in genetic correlation between two traits led to a gain in PA of multi-trait methods in terms of prediction R^2 but the MCP+CTPR performed better than the Lasso+CTPR. It is reverse as compared to the simulation in Figure 2 because this simulation data had a relatively large SNP effects and the MCP+CTPR generally imposes less penalty on large SNP effects than the Lasso+CTPR. Based on recent population genetics studies[1,2], we estimated two theoretical upper bounds of PA for single-trait and multi-trait approaches assuming a genetic correlation of 1 (displayed in grey). Two grey lines represent theoretical upper bound of prediction accuracy for single-trait (lower) and multi-trait approaches (upper). The expected value of prediction R^2 for kth trait is as $E[R^2(y_k,\hat{y}_k)] \approx h_k^2 var(g_k)/\left[var(g_k) + C\cdot\frac{var(y_k)}{N}\right] = h_k^2/\left[1 + C/h_k^2 N\right]$ where h_k^2 is the trait-heritability, g_k is the additive genetic values, y_k and $\hat{y}_k$ are true and predicted phenotype values for kth trait, and C is the number of causal SNPs (Note: this theoretical upper bound of prediction accuracy is achievable if and only if the causal SNPs are used in the prediction model. When causal SNPs are not known a priori, the theoretical upper bound could be lower). Since multi-trait methods used twice as many samples as single-trait methods, the upper bound of PA for multi-trait is

larger than that for single-trait. The difference between theoretical PA and observed PA is large with low genetic correlation between two traits but becomes smaller as the genetic correlation increases.

(b) We next considered the scenarios with 14,800 training samples (7,400 for the primary trait and the other 7,400 for the secondary trait), 5,000 SNPs and 1,000 causal SNPs. "Per-SNP heritability", or the average proportion of phenotypic variance explained by a single SNP, decreases as the number of causal SNPs increases while keeping trait-heritability constant. Doubling the number of causal SNPs (i.e. 1,000) which led to less per-SNP heritability. Less per-SNP heritability resulted in less PA because SNPs associated weakly with the trait tended to be estimated less accurately by the prediction models, which reduced the PA. The smaller per-SNP heritability, the larger relative gain in PA by the multi-trait methods. For example, with 5,000 SNPs, 14,800 samples and 500 causal SNPs, the gain in PA of the multi-trait approach is 10.1% using Lasso and 11.6% using MCP but with 1,000 causal SNPs, the gain in PA is 13.3% using Lasso and 22.2% using MCP (Supplementary Table 2).

Supplementary Figure 2. Effects of proportion of shared causal SNPs on prediction accuracy. We set 2 traits, 14,800 training samples, 5,000 SNPs, 500 casual SNPs, 50% heritability and varied the proportion of shared causal SNPs from 0 to 1 with genetic correlation ρ_{12} = 0.1, 0.5, 0.7 or 0.9.

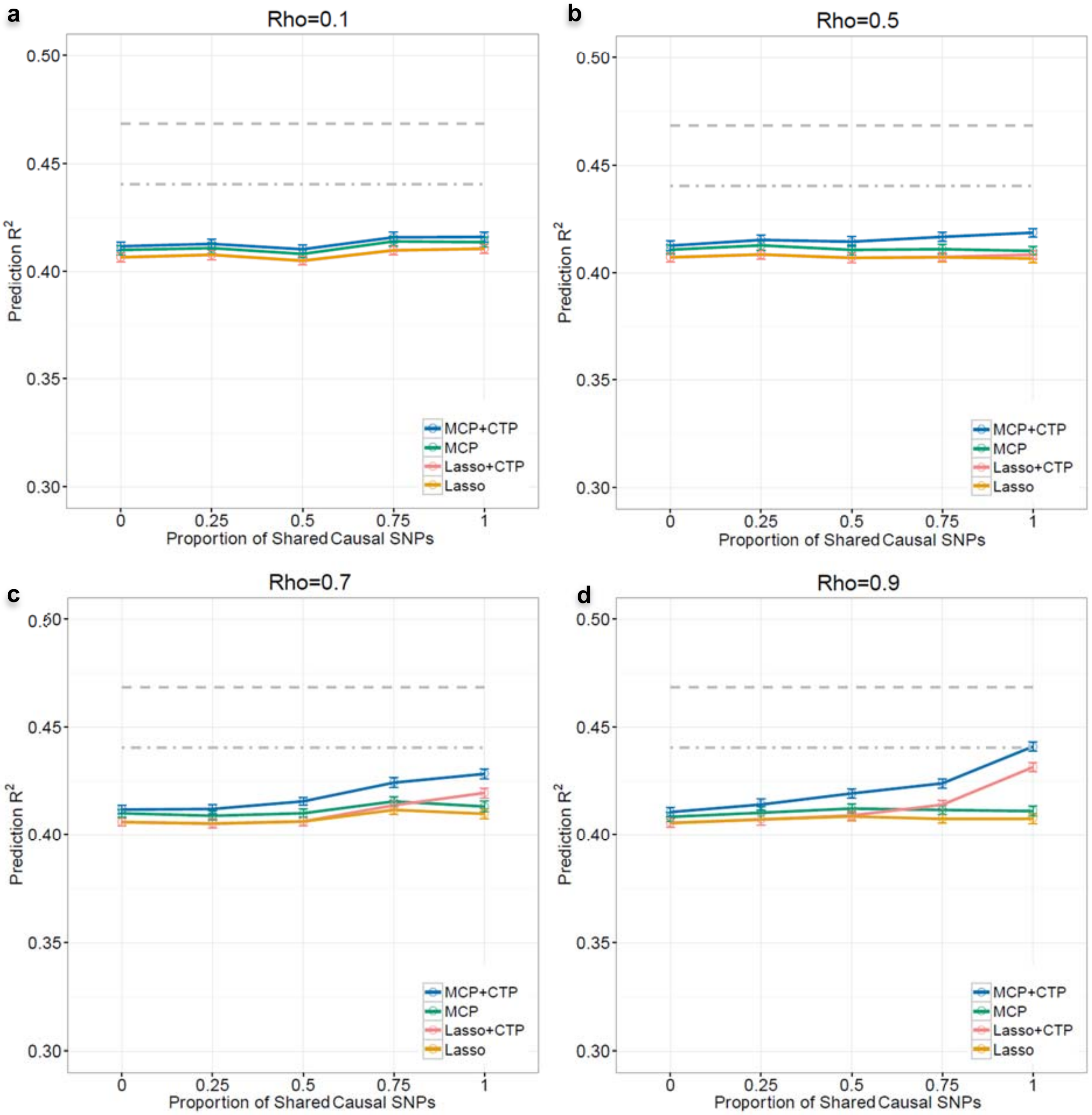

We varied the proportion of shared causal SNPs between two traits using the same simulation setting as for Figure S1a in order to mimic more realistic situations. All error bars represent standard errors of prediction R^2.

(a) Assuming a genetic correlation among the shared causal SNPs of 0.1, there was no apparent gain in PA using multi-trait methods.

(b) (c) (d) As genetic correlation increased to 0.5, 0.7 or 0.9, the improvement in PA at higher proportion of shared causal SNPs among traits became more obvious (Supplementary Table 3).

Supplementary Figure 3. Effects of trait-heritability for primary and secondary traits on prediction accuracy. We set 2 traits, 14,800 training samples, 5,000 SNPs, 500 casual SNPs and varied trait-heritability to h^2=(h_1^2, h_2^2)= (50%,25%), (50%,75%), (25%,25%) or (75%,75%) where h_k^2 is the pre-set heritability for the kth trait.

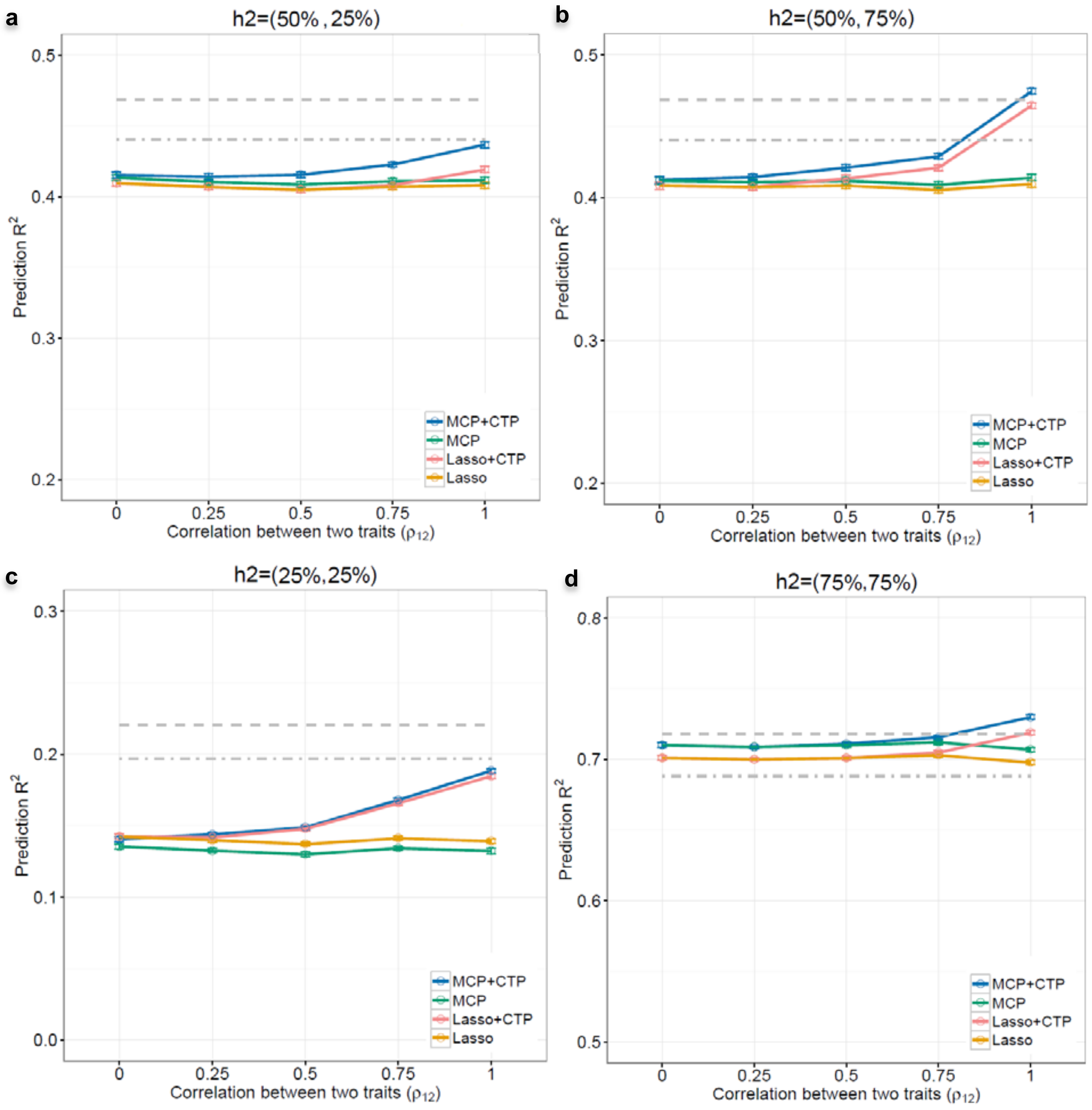

To demonstrate the effect of trait-heritability on PA, we considered the setting where the training sample size, the number of SNPs and the number of causal SNPs were 14,800, 5,000 and 500, respectively. All error bars represent standard errors of prediction R^2.

(a) (b) Compared to the previous settings with 50% heritability for both traits in Figure S1a, we changed the heritability of the secondary trait to 25% or 75%. While the PA of the single-trait approaches were 0.407 for Lasso and 0.411 for MCP, the PA of the multi-trait methods were 0.408, 0.423 with the heritability of 25% for the secondary trait but 0.421, 0.429 with the heritability of 75% and genetic correlation=0.75 (Supplementary Table 4). The results showed

that larger heritability for secondary traits help PA of the primary trait even if the genetic correlation between traits remains the same.

(c) (d) We examined the heritability of 25% or 75% for both traits. While overall patterns were quite similar to the results with the heritability of 50%, the PA of single-trait methods and multi-trait methods ranged between 0.137 and 0.188 with a heritability of 25% and between 0.699 and 0.730 with a heritability of 75% (Supplementary Table 4). The results showed that larger trait-heritability of both traits helps PA of the primary trait.

Supplementary Figure 4. Effects of number of traits on prediction accuracy. We simulated multiple phenotypes, each with 2,000 training samples, 5,000 SNPs, 500 casual SNPs, 50% heritability and varied number of trait from 2 to 7 with genetic correlation ρ_{ij}= 0.1, 0.5, 0.7 or 0.9 ($i \neq j$ $i \neq 1, j \neq 1$), ρ_{1i} = ρ_{i1}= 0.5.

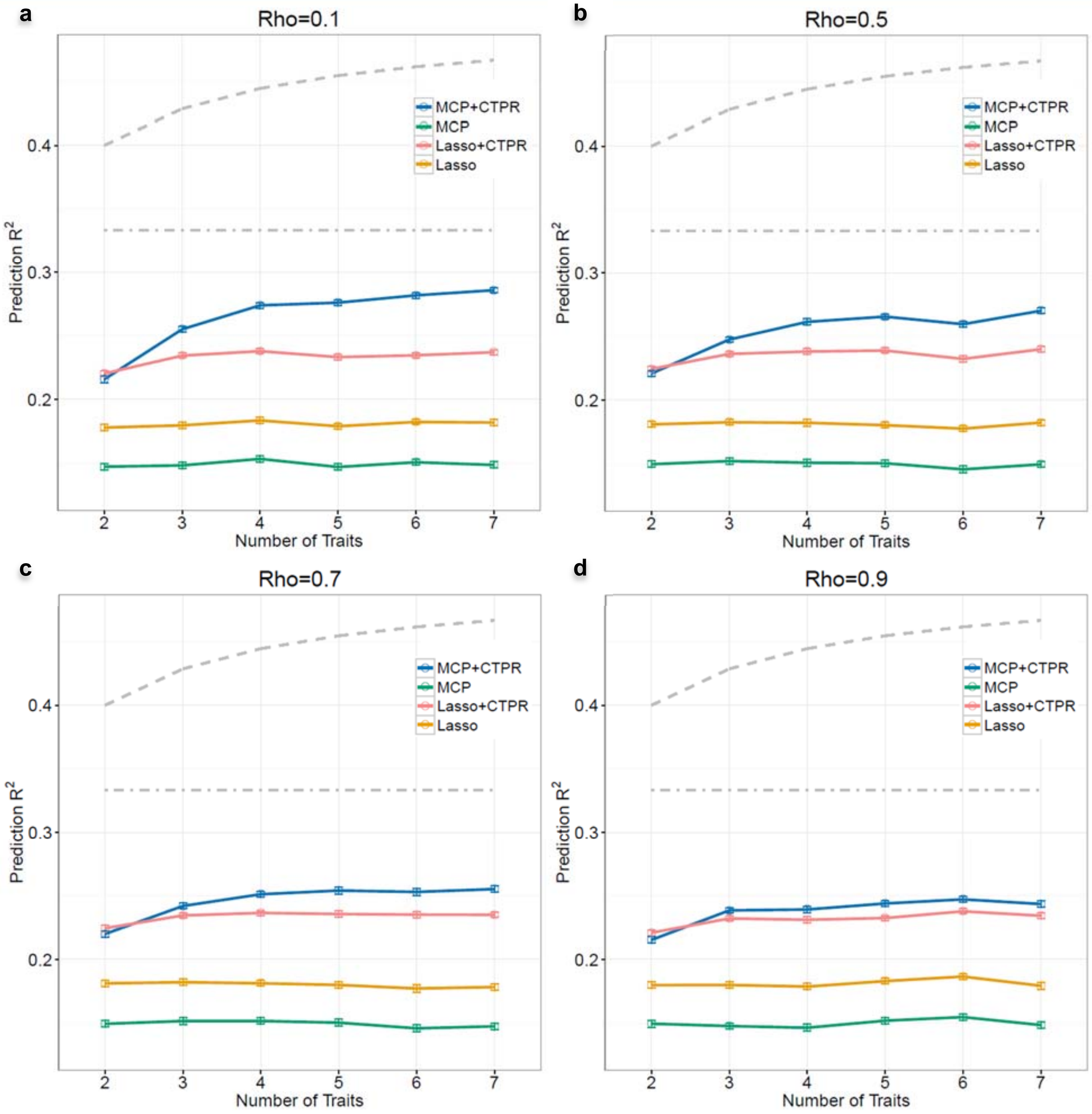

To assess the effect of number of secondary traits on PA of the primary trait, we simulated multiple phenotypes, each with 2,000 samples. The genetic correlation between the primary trait and each of the secondary trait was fixed to 0.5 and the genetic correlation among secondary traits varied from 0.1 to 0.9. We evaluated how the number of secondary traits and genetic correlation among secondary traits would affect the PA for the primary trait. All error bars represent standard errors of prediction R^2.

(a) (b) (c) (d) With a genetic correlation of 0.1 among secondary traits, the PA was 0.220 (MCP+CTPR) when including two traits and 0.286 for seven traits. The PA increased as more

traits were included. With a genetic correlation of 0.9 among secondary traits, the PA was 0.220 (MCP+CTPR) for two traits and 0.244 for seven traits (Supplementary Table 5). The results showed that including more secondary traits that were less genetically correlated with each other would increase the PA for the primary trait.

Supplementary Figure 5. Comparisons of the prediction accuracy of the proposed methods (MCP+CTPR, Lasso+CTPR) using individual-level genotypes, true coefficients and summary statistics.

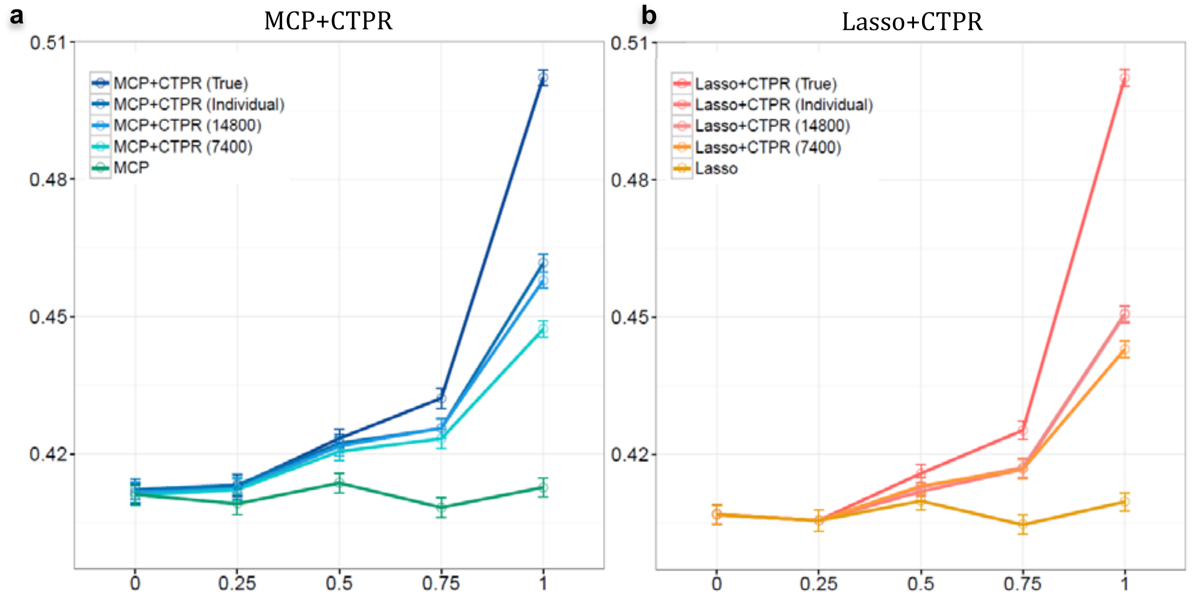

To utilize summary statistics from published GWAS results for improving PA of the primary trait, we implemented a novel cross-trait penalty function that can exploit fixed SNP effects of the secondary trait and evaluated its performance systematically using simulations. For the secondary trait, we used true SNP effects as well as estimated SNP effects via single SNP linear regression based on 7,400 and 14,800 samples, respectively. The prediction R^2 using three summary data (i.e. true, 7,400 and 14,800) and individual-level data were compared after averaging over 100 replications. All error bars represent standard errors of prediction R^2.

(a) (b) The use of true SNP coefficients for the secondary trait produced the best PA across all genetic correlation settings. We also observed that individual-level data of 7,400 samples for the secondary trait gave slightly better PA than the use of summary statistics based on 14,800 and 7,400 samples. For example, at genetic correlation of 0.75, the prediction R^2 using Lasso+CTPR (MCP+CTPR) was 0.425 (0.432) with true SNP effects, and 0.417 (0.426) with summary statistics based on 14,800 samples, and 0.416 (0.423) with summary statistics based on 7,400 samples (Supplementary Table 6). Since the use of true SNP effects gives the best PA and PA increases as sample size of the summary statistics increases, it would be expected that use of summary statistics from external large-scale GWAS studies could outperform use of individual-level data of the secondary trait (usually with smaller sample size). All published GWAS summary statistics for related secondary traits could then be used to improve the prediction model of the primary trait. Computational advantage using summary statistics makes it feasible to incorporate a large number of published GWAS studies in building the prediction model for the primary trait.

Abbreviations: True=true coefficients for the secondary trait, Individual= individual-level genotypes, 7400=summary statistics computed with 7,400 samples, 14,800=summary statistics computed with 14,800 samples.

Supplementary Figure 6. Performance evaluation of coordinate decent algorithm with 14,800 training samples, 5,000 SNPs and genetic correlation=0.75. (a) Plot for number of non-zero $\widehat{\beta}$ vs. number of causal SNPs. (b) Plot for λ_1 estimates vs. number of causal SNPs. (c) Plot for λ_2 estimates vs. genetic correlation between two traits (ρ_{12}).

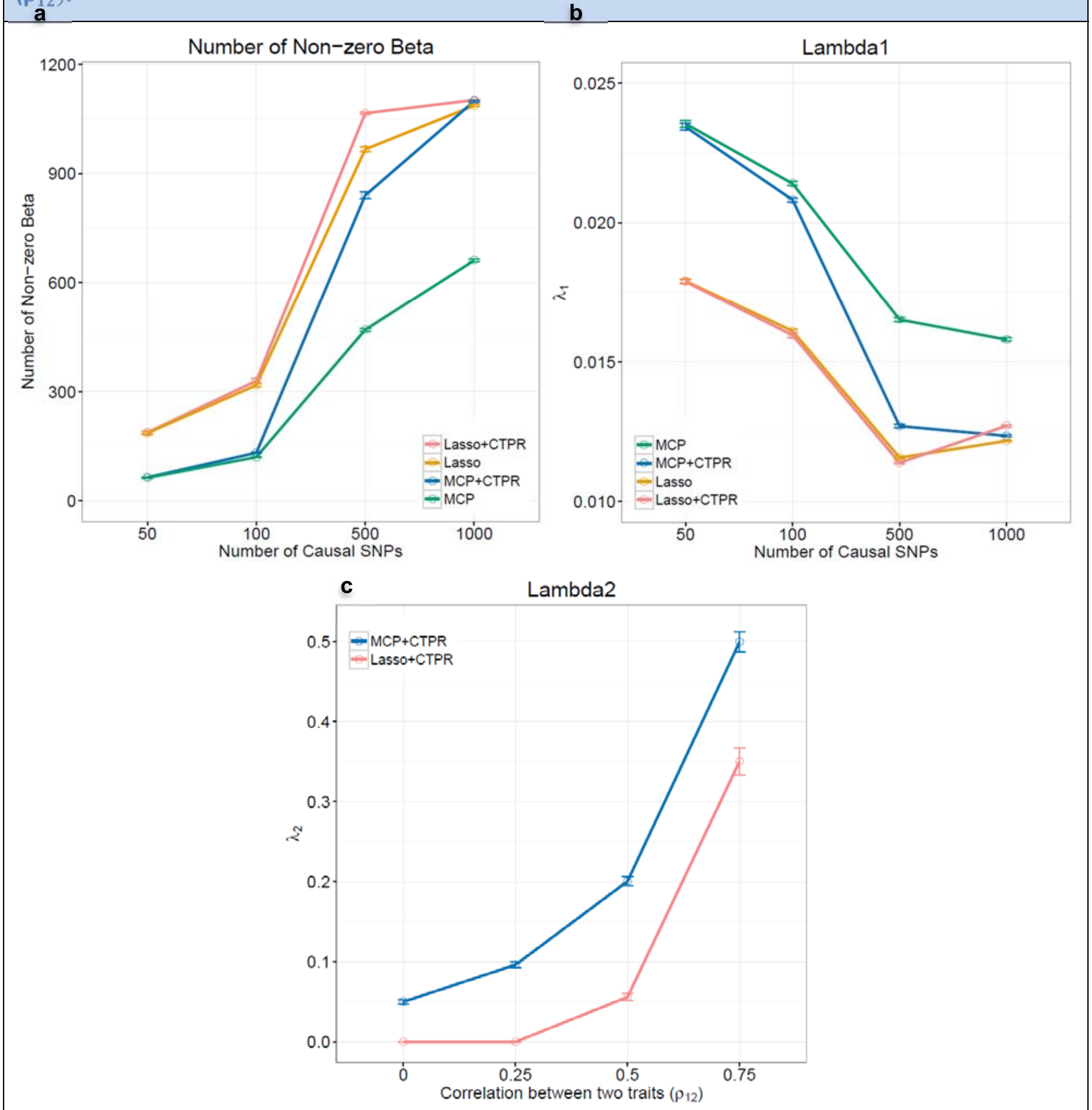

We implemented coordinate descent algorithm for obtaining the estimates for SNP effects ($\widehat{\boldsymbol{\beta}}$) in the proposed prediction model. Below we evaluated how well the model fits the data with one of our simulation scenarios where the number of training samples, number of SNPs and genetic correlation are 14,800, 5,000 and 0.75. All error bars represent standard errors of prediction R^2.

(a) (b) We varied the number of causal SNPs from 50 to 1,000 and summarized the number of estimated non-zero SNP effects and the tuning parameter for sparsity penalty (λ_1) by averaging

over 100 replications. As the number of causal SNPs increased, the number of estimated non-zero SNP effects increased but the sparsity tuning parameter (λ_1) decreased. For example, with 50 causal SNPs, we had 63.27 (or 185.96) non-zero SNP effects and λ_1=0.0235 (or 0.0179) using MCP (or Lasso) but, with 1,000 causal SNPs, we had 660.97 (or 1086.56) non-zero SNP effects and λ_1=0.0157 (or 0.0121). Multi-trait methods tended to have more non-zero SNP effects than single-trait methods because of the induced smoothness of the coefficients from cross-trait penalty function. Since small (or large) λ_1 may be selected with large (or small) number of non-zero SNP effects, the $\lambda_1$estimates showed the opposite pattern to the number of non-zero SNP effects.

(c) We varied the genetic correlation from 0 to 0.75 with 500 causal SNPs and reported the average tuning parameters for cross-trait penalty (λ_2) over 100 replications. As expected, larger genetic correlation led to larger λ_2 for cross-trait penalty. We then computed the slopes of the regression of phenotypic values on the predicted ones within the same simulation setting. Most slopes were close to 1 for both single-trait and multi-trait methods (range=0.97-1.31) in Supplementary Table 2.

Supplementary Figure 7. Scatter plots for phenotypic correlation between height (HGT) vs. body mass index (BMI) based on original and age, sex-adjusted phenotypic values in UK Biobank data and NHS/HPFS/PHS Cohort.

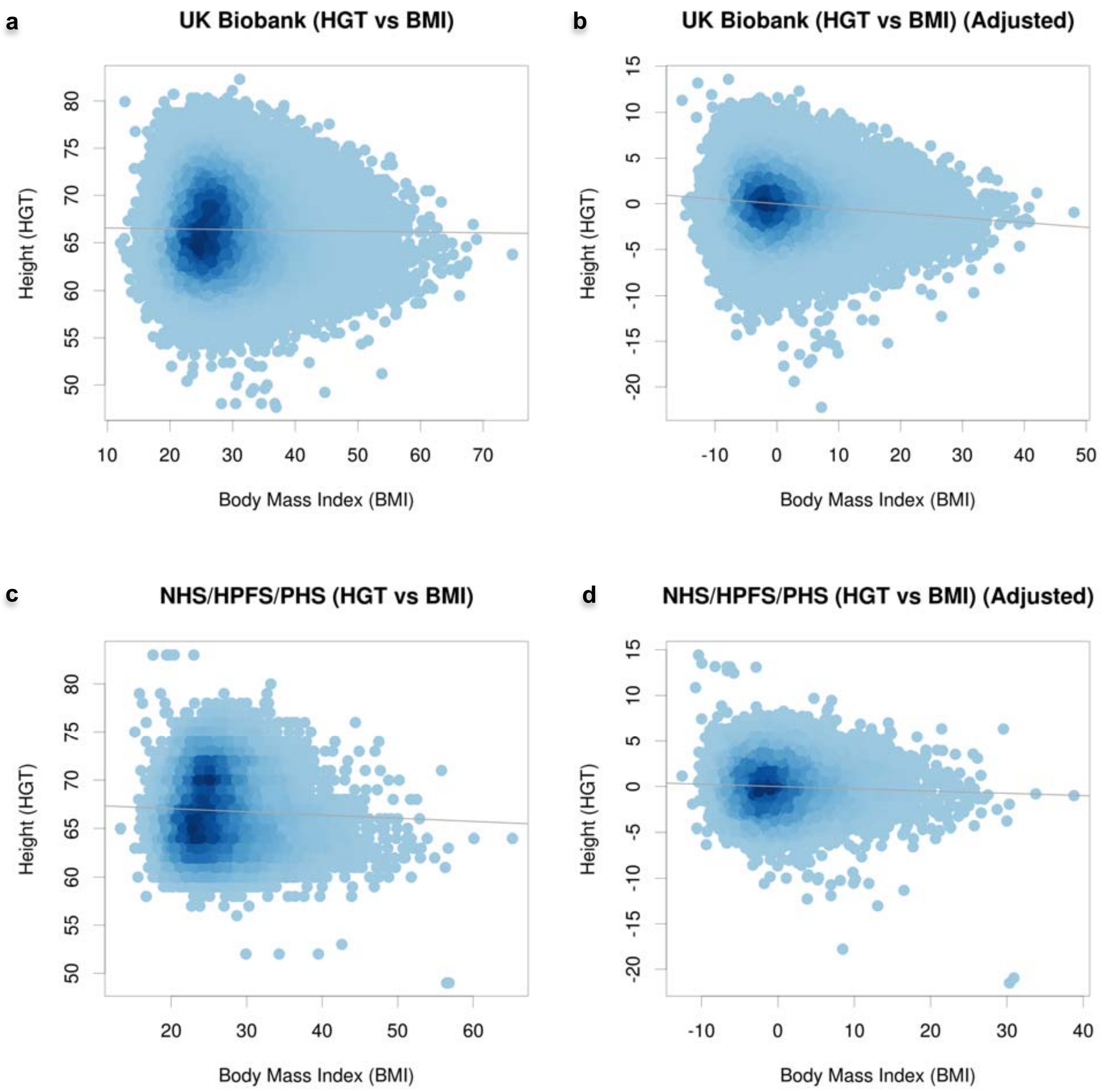

(a) (b) The phenotypic correlations between HGT and BMI were computed in UK Biobank with and without adjusting for age and sex. After adjusting for age and sex, HGT and BMI were negatively correlated.

(c) (d) The phenotypic correlations between HGT and BMI were computed in NHS/HPFS/PHS cohort with and without adjusting for age and sex. After adjusting for age and sex, HGT and BMI were negatively correlated.

Supplementary Figure 8. Scatter plots for phenotypic correlation between height (HGT) vs. other phenotypes (HIP, WST and WHR) based on original and age, sex-adjusted phenotypic values in UK Biobank data.

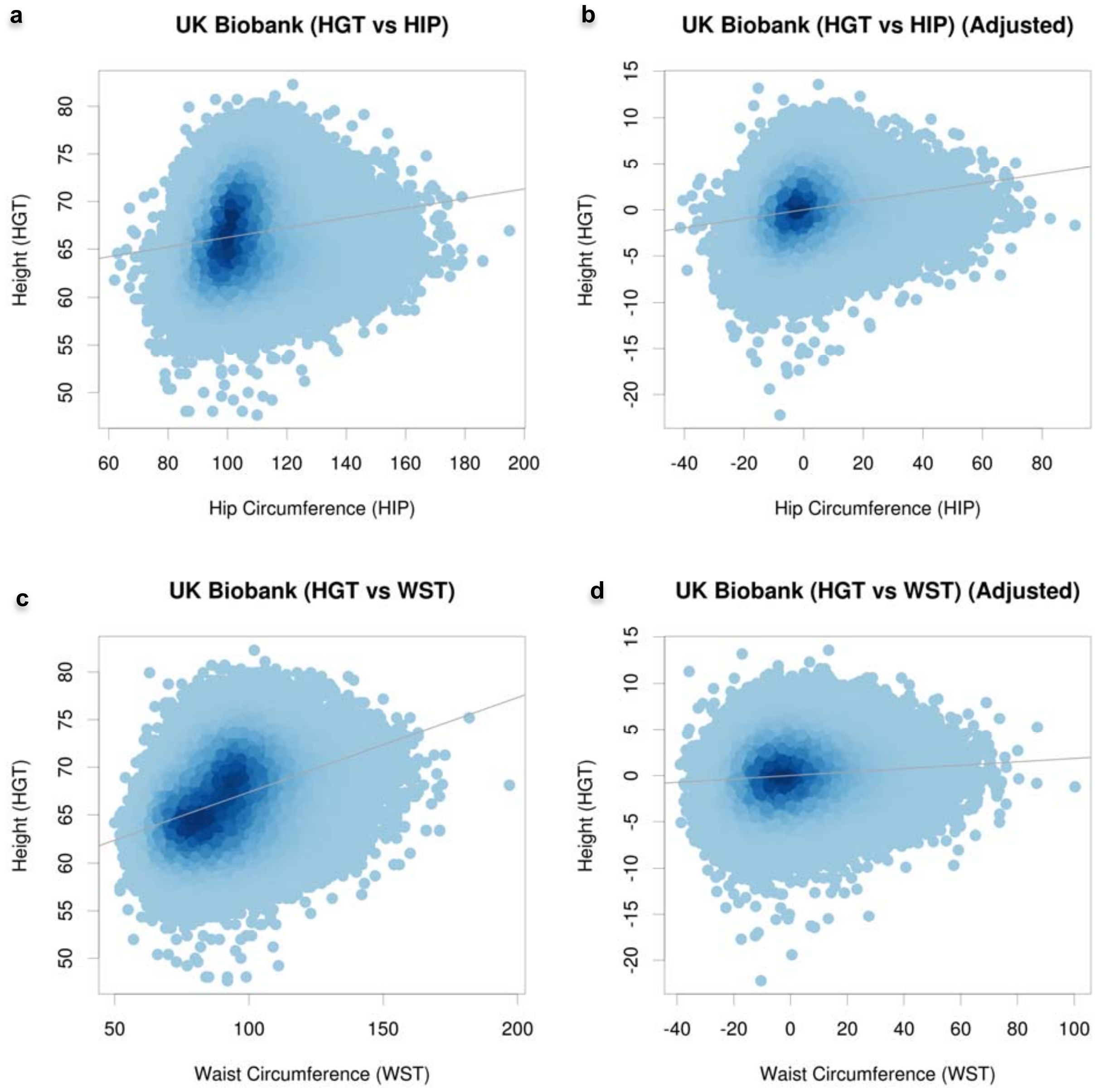

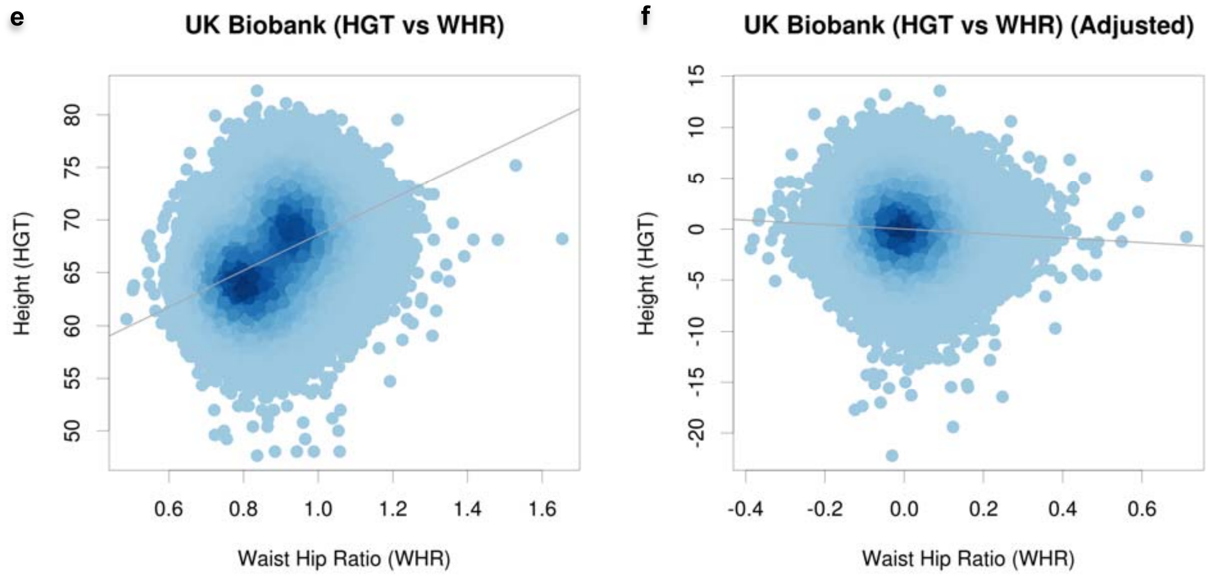

(a) (b) The phenotypic correlations between HGT and HIP were computed in UK Biobank with and without adjusting for age and sex. After adjusting for age and sex, HGT and BMI were positively correlated.

(c) (d) The phenotypic correlations between HGT and WST were computed in UK Biobank with and without adjusting for age and sex. After adjusting for age and sex, HGT and WST were positively correlated.

(e) (f) The phenotypic correlations between HGT and WHR were computed in UK Biobank with and without adjusting for age and sex. After adjusting for age and sex, HGT and WHR were negatively correlated.

Supplementary Figure 9. Effect of sample size and trait-heritability on prediction accuracy using Lasso or Ridge based methods with 5,000 SNPs. (a) We considered three different training sample sizes (N = 3,700 / 7,400 / 14,800) with 50% trait-heritability. (b) We examined three different trait-heritability levels ($\mathbf{h}^2$ = 20% / 40% / 60%) with 7,400 samples.

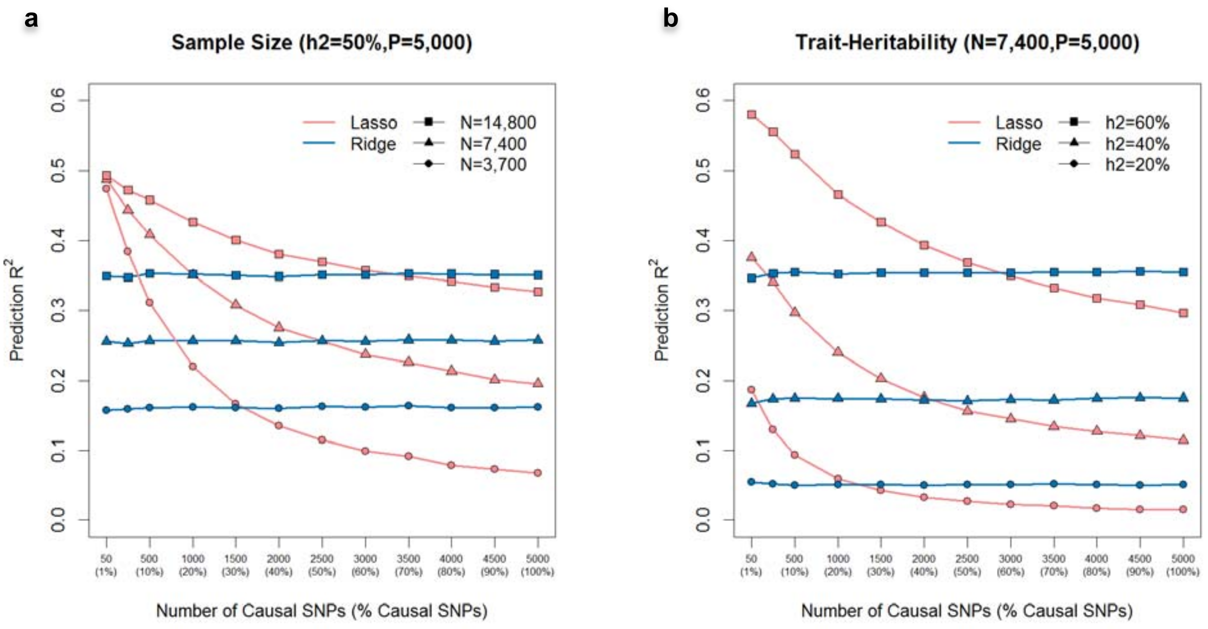

To demonstrate how the power to detect causal SNPs would affect the relative performance between our penalized regression based methods (Lasso) and GBLUP (or Ridge) based methods, we conducted simulations with 5,000 SNPs using 'glmnet' package (version 2.0.1) in R. We considered three different sample sizes (i.e. 3,700, 7,400, 14,800) with 50% trait-heritability and next examined three different trait-heritability levels (i.e. 20%, 40% or 60%) with 7,400 samples while we varied the proportion of causal SNPs from 1% to 100%. The prediction R^2 were averaged over 100 replications. We confirmed that Lasso results using our software were the same as Lasso using 'glmnet' and GBLUP results were the same as Ridge using 'glmnet'. For given sample size and given total trait-heritability, the Ridge/GBLUP based method is always the best for the 100% infinitesimal scenario. However, when the true genetic architecture is deviated from 100% infinitesimal model, the Lasso based method becomes better. Importantly, the crossing point of the Lasso (red) and the Ridge/GBLUP (blue) lines moves toward 100% causal SNPs as the power to detect causal SNP increases (i.e. by increasing sample size or increasing trait-heritability). For example, for a training sample size of 14,800, the Lasso based method becomes better than GBLUP based method even if more than 65% of all SNPs are causal.

(a) We considered three different training sample sizes (N = 3,700 / 7,400 / 14,800) with 50% trait-heritability to compare Lasso vs. Ridge based methods.

(b) We examined three different trait-heritability levels ($\mathbf{h}^2$ = 20% / 40% / 60%) with 7,400 samples to compare Lasso vs. Ridge based methods.

Supplementary Figure 10. Effect of number of SNPs on prediction accuracy using Lasso vs Ridge based methods with 40% trait-heritability. (a) We considered four different number of SNPs (P = 5,000 / 10,000 / 20,000 / 40,000) with 11,473 samples. (b) We considered four different number of SNPs (P = 5,000 / 10,000 / 20,000 / 40,000) with 22,946 samples.

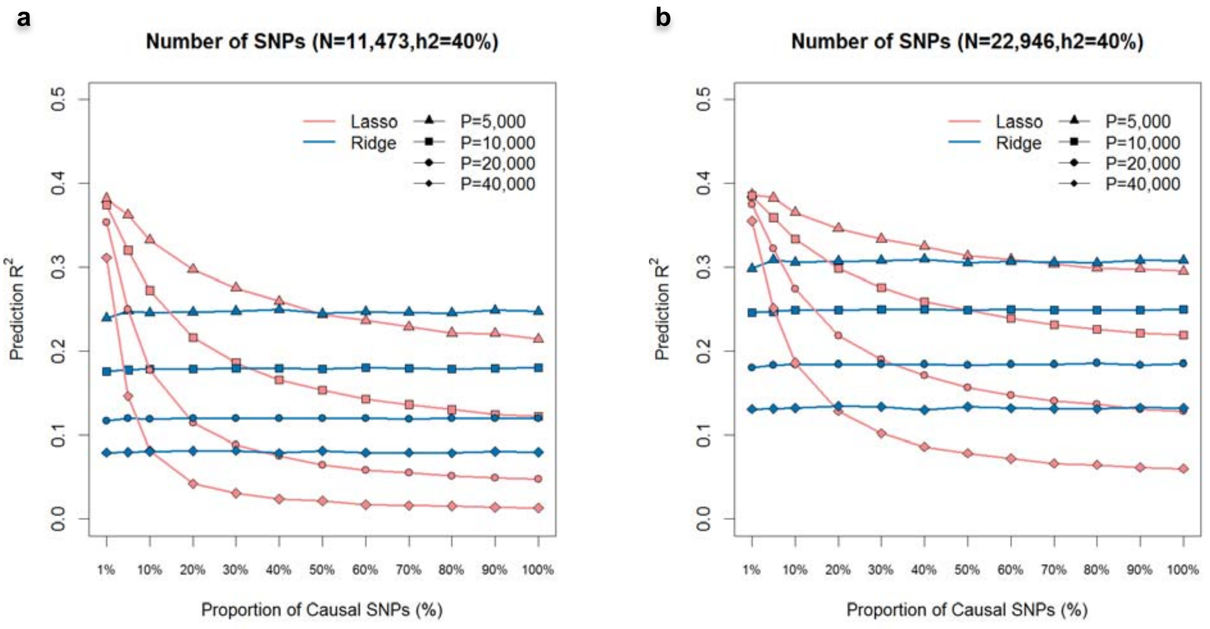

We examined the effect of large number of SNPs (i.e. low prediction power) on performance comparison between Lasso and Ridge/GBLUP based methods. We considered four different numbers of SNPs (i.e. 5,000, 10,000, 20,000 and 40,000) with 40% trait-heritability and 11,473 samples while we varied the proportion of causal SNPs from 1% to 100%. As expected, the crossing point of Lasso and Ridge/GBLUP moves toward less causal SNP models as more SNPs are included. For example, the crossing point was 50% with 5,000 SNPs while it became 11% with 40,000 SNPs. However, it moves toward more causal SNP models with larger sample sizes.

(a) We considered four different number of SNPs (P = 5,000 / 10,000 / 20,000 / 40,000) with 11,473 samples to compare predictive performance between Lasso and Ridge based methods.

(b) We considered four different number of SNPs (P = 5,000 / 10,000 / 20,000 / 40,000) with 22,946 samples to compare predictive performance between Lasso and Ridge based methods.

Supplementary Figure 11. Simulation results using PRS, LDpred, MTAG, MTGBLUP and CTPR methods when genetic correlation between two traits, ρ =0.15. (a) Effect of number of traits (T) on prediction accuracy with N=30K and ρ=0.15. (b) Effect of sample size on prediction accuracy with T=2 and ρ=0.15.

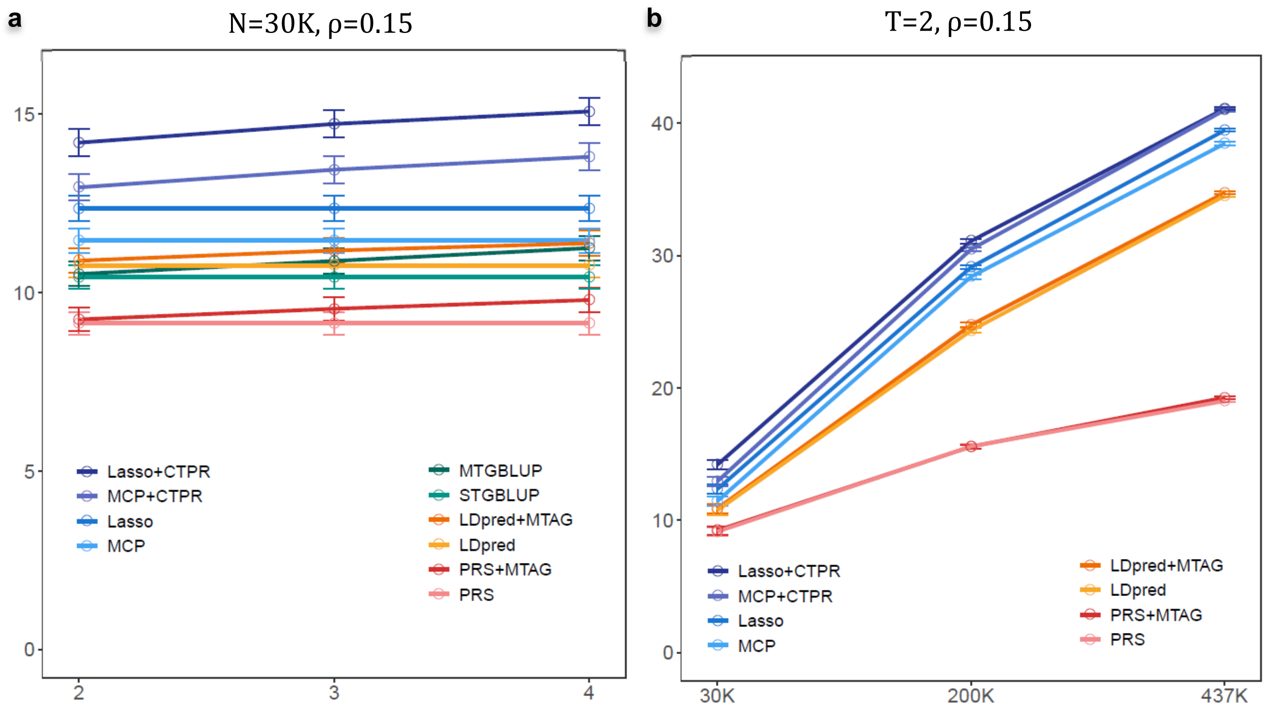

To more closely match the genetic correlation obtained in the real data, we conducted additional simulations with the genetic correlation among traits=0.15. We first considered the scenario with 30K training samples, two traits and genetic correlation=0.15. Because generic correlation between two traits is smaller than the previous simulation settings in Figure 2a, a gain in PA of multi-trait methods over single-trait methods is smaller. And then we included more traits from 2 to 4 while the genetic correlation between the primary trait and each of the secondary traits=0.15 and the genetic correlation among secondary traits=0. The PA of multi-trait methods increased as more traits were included but a gain in PA over single-trait methods decreased when compared to results in Figure 2b. We next increased the sample size from 30K to 437K and found that the PA of both single-trait and multi-trait methods adequately increased but the gap between them is smaller than the results in Figure 2c. All error bars represent standard errors of prediction R^2.

Supplementary Table 1. Prediction R^2 and mean squared error (MSE) with different sample sizes (N=30K,200K,437K) and number of traits (T=2,3,4) and genetic correlations (ρ=0.25,0.5,0.75) using LDpred, MTAG, STGBLUP, MTGBLUP and CTPR methods.

N	#Traits	ρ	Method	R^2	MSE	Slope
30K	2	0.25	PRS	0.0914	8154.01	-0.0094
			LDpred	0.1076	1.4363	0.9077
			STGBLUP	0.1043	0.8869	0.9854
			MCP	0.1145	0.8867	0.9812
			LAS	0.1235	0.8878	1.4261
			PRS+MTAG	0.0926	6602.41	-0.0095
			LDpred+MTAG	0.1094	1.2972	0.8637
			MTGBLUP	0.1057	0.8855	0.9910
			MCP+CTPR	0.1345	0.8739	1.43313
			LAS+CTPR	0.1425	0.8549	1.29360
30K	2	0.5	PRS	0.0914	8154.01	-0.0094
			LDpred	0.1076	1.4363	0.9077
			STGBLUP	0.1043	0.8869	0.9854
			MCP	0.1145	0.8867	0.9812
			LAS	0.1235	0.8878	1.4261
			PRS+MTAG	0.0943	7349.59	-0.0097
			LDpred+MTAG	0.1123	1.4291	0.8469
			MTGBLUP	0.1086	0.8826	0.9953
			MCP+CTPR	0.1393	0.8672	1.3437
			LAS+CTPR	0.1470	0.8500	1.27678
30K	2	0.75	PRS	0.0914	8154.01	-0.0094
		1	LDpred	0.1076	1.4363	0.9077
			STGBLUP	0.1043	0.8869	0.9854
			MCP	0.1145	0.8867	0.9812
			LAS	0.1235	0.8878	1.4261
			PRS+MTAG	0.0975	5983.37	-0.0100
			LDpred+MTAG	0.1170	1.1912	0.8261
			MTGBLUP	0.1130	0.8782	1.0034
			MCP+CTPR	0.1441	0.8599	1.2654
			LAS+CTPR	0.1529	0.8440	1.2651
30K	3	0.5	PRS	0.0914	8154.01	-0.0094
			LDpred	0.1076	1.4363	0.9077
			STGBLUP	0.1043	0.8869	0.9854
			MCP	0.1145	0.8867	0.9812
			LAS	0.1235	0.8878	1.4261
			PRS+MTAG	0.0988	5564.60	-0.0114
			LDpred+MTAG	0.1183	1.1467	0.8350

			MTGBLUP	0.1158	0.8643	1.0024
			MCP+CTPR	0.1447	0.8802	1.8260
			LAS+CTPR	0.1515	0.8564	1.5214
30K	4	0.5	PRS	0.0914	8154.01	-0.0094
			LDpred	0.1076	1.4363	0.9077
			STGBLUP	0.1043	0.8869	0.9854
			MCP	0.1145	0.8867	0.9812
			LAS	0.1235	0.8878	1.4261
			PRS+MTAG	0.0998	5395.40	-0.0125
			LDpred+MTAG	0.1194	1.1325	0.8435
			MTGBLUP	0.1164	0.8536	1.0064
			MCP+CTPR	0.1475	0.8346	1.2896
			LAS+CTPR	0.1540	0.8246	1.2984
200K	2	0.5	PRS	0.1559	4129.56	-0.0163
			LDpred	0.2435	2.5768	0.6544
			MCP	0.2842	0.7645	0.4763
			LAS	0.2913	0.7404	0.6059
			PRS+MTAG	0.1554	2922.19	-0.0163
			LDpred+MTAG	0.2499	2.9502	0.6424
			MCP+CTPR	0.3094	0.7494	0.5328
			LAS+CTPR	0.3118	0.7295	0.6295
437K	2	0.5	PRS	0.1902	3270.83	-0.0179
			LDpred	0.3453	141.7601	0.1797
			MCP	0.3848	0.7292	0.5584
			LAS	0.3948	0.6948	0.6984
			PRS+MTAG	0.1933	2609.21	-0.0179
			LDpred+MTAG	0.3483	54.9642	0.1760
			MCP+CTPR	0.4128	0.6948	0.6948
			LAS+CTPR	0.4148	0.6489	0.7948

Abbreviations: #Traits=number of traits, ρ=genetic correlation between two traits, R^2=prediction accuracy, Slope=slope of the regression of phenotypic values on the predicted ones

Supplementary Table 2. Prediction R^2 and mean squared error (MSE) with number of causal SNPs=500 or 1000 and 2 traits, 14,800 training samples, 5,000 SNPs, h^2= (0.5, 0.5) and 100 replicates.

# Causal	Method	ρ_{12}	R^2	SE(R^2)	MSE	SE(MSE)	Slope	# Nz β	λ_1	λ_2
500	Lasso	0	0.4109	0.0021	0.6019	0.0013	1.1826	985.11	0.0114	.
		0.25	0.4093	0.0020	0.6021	0.0013	1.1802	987.36	0.0114	.
		0.5	0.4069	0.0025	0.6008	0.0013	1.1829	980.25	0.0115	.
		0.75	0.4074	0.0022	0.6035	0.0012	1.1861	966.5	0.0116	.
		1	0.4102	0.0020	0.6039	0.0014	1.1895	974.97	0.0115	.
	Lasso+CTPR	0	0.4109	0.0021	0.6019	0.0013	1.1826	985.11	0.0114	0.0000
		0.25	0.4093	0.0020	0.6021	0.0013	1.1802	987.36	0.0114	0.0000
		0.5	0.4091	0.0024	0.6014	0.0013	1.2128	1021.7	0.0113	0.0560
		0.75	0.4193	0.0021	0.6011	0.0012	1.2751	1065.56	0.0114	0.3502
		1	0.4514	0.0019	0.5779	0.0012	1.3139	1023.7	0.0124	8.9349
	MCP	0	0.4147	0.0022	0.5884	0.0013	0.9788	474.57	0.0164	.
		0.25	0.4130	0.0021	0.5890	0.0011	0.9733	480.98	0.0164	.
		0.5	0.4104	0.0025	0.5878	0.0012	0.9777	473.46	0.0164	.
		0.75	0.4106	0.0022	0.5904	0.0012	0.9784	469.64	0.0165	.
		1	0.4145	0.0021	0.5892	0.0013	0.9812	476.5	0.0164	.
	MCP+CTPR	0	0.4157	0.0022	0.5875	0.0013	1.0192	534.24	0.0156	0.0501
		0.25	0.4170	0.0021	0.5852	0.0011	1.0290	582.69	0.0150	0.0962
		0.5	0.4186	0.0025	0.5798	0.0012	1.0362	663.43	0.0142	0.2006
		0.75	0.4275	0.0021	0.5733	0.0010	1.0107	840.07	0.0127	0.4993
		1	0.4624	0.0019	0.5408	0.0010	0.9957	1020.49	0.0119	11.1990
1000	Lasso	0	0.3444	0.0021	0.6705	0.0013	1.2353	1085.25	0.0122	.
		0.25	0.3482	0.0018	0.6691	0.0015	1.2323	1093.13	0.0121	.
		0.5	0.3442	0.0017	0.6687	0.0014	1.2317	1087.72	0.0121	.
		0.75	0.3438	0.0018	0.6698	0.0014	1.2361	1086.56	0.0122	.
		1	0.3425	0.0016	0.6687	0.0014	1.2276	1087.82	0.0122	.
	Lasso+CTPR	0	0.3444	0.0021	0.6705	0.0013	1.2353	1085.25	0.0122	0.0000
		0.25	0.3483	0.0018	0.6692	0.0015	1.2352	1094.49	0.0121	0.0031
		0.5	0.3477	0.0017	0.6701	0.0015	1.2841	1107.35	0.0122	0.0650
		0.75	0.3602	0.0017	0.6699	0.0015	1.3959	1101.67	0.0127	0.3116
		1	0.3881	0.0015	0.6508	0.0013	1.4693	1073.77	0.0138	4.1057
	MCP	0	0.3327	0.0022	0.6708	0.0014	0.9477	656.69	0.0158	.
		0.25	0.3360	0.0020	0.6702	0.0016	0.9474	657.78	0.0158	.
		0.5	0.3321	0.0019	0.6697	0.0014	0.9485	652.99	0.0159	.
		0.75	0.3315	0.0020	0.6707	0.0015	0.9483	660.97	0.0158	.
		1	0.3310	0.0017	0.6700	0.0015	0.9402	663.06	0.0157	.
	MCP+CTPR	0	0.3431	0.0020	0.6598	0.0013	1.0332	930.05	0.0130	0.1544
		0.25	0.3543	0.0019	0.6509	0.0015	1.0334	1038.15	0.0123	0.2392
		0.5	0.3607	0.0017	0.6403	0.0013	1.0250	1089.53	0.0121	0.3557
		0.75	0.3758	0.0018	0.6254	0.0014	1.0185	1098.9	0.0123	0.6157
		1	0.4047	0.0015	0.5948	0.0012	1.0167	1080.52	0.0130	4.3590

Abbreviations: # Causal=number of causal SNPs, ρ_{12}=genetic correlation between two traits, #Nz β=number of estimated nonzero SNP effects

Supplementary Table 3. Prediction R^2 and mean squared error (MSE) with ρ_{12} = 0.1, 0.5, 0.7 or 0.9 and 2 traits, 14,800 training samples, 5,000 SNPs, h^2= (0.5, 0.5) and 100 replicates.

ρ_{12}	Method	%S	R^2	SE(R^2)	MSE	SE(MSE)	Slope	# Nz β	λ_1	λ_2
0.1	Lasso	0	0.4066	0.0021	0.6030	0.0014	1.1867	984.0	0.0115	.
		0.25	0.4078	0.0022	0.6024	0.0014	1.1866	973.2	0.0115	.
		0.5	0.4050	0.0019	0.6015	0.0012	1.1836	973.6	0.0115	.
		0.75	0.4098	0.0023	0.6019	0.0012	1.1751	987.4	0.0114	.
		1	0.4106	0.0021	0.6028	0.0012	1.1845	986.7	0.0114	.
	Lasso+CTPR	0	0.4066	0.0021	0.6030	0.0014	1.1867	984.0	0.0115	0.0000
		0.25	0.4078	0.0022	0.6024	0.0014	1.1866	973.2	0.0115	0.0000
		0.5	0.4050	0.0019	0.6015	0.0012	1.1836	973.6	0.0115	0.0000
		0.75	0.4098	0.0023	0.6019	0.0012	1.1751	987.4	0.0114	0.0000
		1	0.4106	0.0021	0.6028	0.0012	1.1845	986.7	0.0114	0.0000
	MCP	0	0.4100	0.0021	0.5897	0.0013	0.9787	475.1	0.0164	.
		0.25	0.4109	0.0023	0.5894	0.0014	0.9773	471.4	0.0165	.
		0.5	0.4082	0.0019	0.5890	0.0012	0.9765	468.9	0.0165	.
		0.75	0.4140	0.0023	0.5890	0.0012	0.9718	475.8	0.0164	.
		1	0.4136	0.0022	0.5900	0.0013	0.9801	469.6	0.0165	.
	MCP+CTPR	0	0.4117	0.0021	0.5883	0.0013	1.0364	544.0	0.0154	0.0605
		0.25	0.4128	0.0023	0.5877	0.0014	1.0311	541.3	0.0155	0.0599
		0.5	0.4103	0.0019	0.5868	0.0012	1.0278	539.9	0.0155	0.0605
		0.75	0.4158	0.0024	0.5869	0.0012	1.0213	548.2	0.0154	0.0627
		1	0.4160	0.0022	0.5876	0.0013	1.0262	542.2	0.0154	0.0620
0.5	Lasso	0	0.4073	0.0022	0.6030	0.0013	1.1878	977.5	0.0115	.
		0.25	0.4086	0.0023	0.6013	0.0014	1.1818	985.6	0.0114	.
		0.5	0.4070	0.0024	0.6031	0.0011	1.1877	974.0	0.0115	.
		0.75	0.4073	0.0021	0.6048	0.0014	1.1863	978.0	0.0115	.
		1	0.4067	0.0019	0.6023	0.0011	1.1868	972.9	0.0116	.
	Lasso+CTPR	0	0.4073	0.0022	0.6030	0.0013	1.1878	977.5	0.0115	0.0000
		0.25	0.4086	0.0023	0.6013	0.0014	1.1818	985.6	0.0114	0.0000
		0.5	0.4070	0.0024	0.6031	0.0011	1.1877	974.0	0.0115	0.0000
		0.75	0.4074	0.0021	0.6050	0.0014	1.1897	982.7	0.0115	0.0055
		1	0.4085	0.0019	0.6027	0.0011	1.2098	1019.9	0.0113	0.0488
	MCP	0	0.4108	0.0022	0.5895	0.0012	0.9798	475.7	0.0164	.
		0.25	0.4128	0.0024	0.5876	0.0013	0.9778	477.4	0.0164	.
		0.5	0.4107	0.0024	0.5894	0.0011	0.9801	473.2	0.0164	.
		0.75	0.4110	0.0022	0.5913	0.0014	0.9782	476.8	0.0164	.
		1	0.4103	0.0020	0.5889	0.0011	0.9776	472.4	0.0165	.
	MCP+CTPR	0	0.4127	0.0022	0.5879	0.0012	1.0355	549.3	0.0154	0.0606
		0.25	0.4153	0.0023	0.5852	0.0013	1.0285	557.1	0.0153	0.0658
		0.5	0.4145	0.0024	0.5862	0.0011	1.0419	578.1	0.0150	0.0978
		0.75	0.4168	0.0022	0.5859	0.0014	1.0423	612.1	0.0147	0.1330

		1	0.4187	0.0020	0.5808	0.0011	1.0377	647.8	0.0143	0.1878
0.7	Lasso	0	0.4058	0.0019	0.6063	0.0013	1.1836	982.1	0.0115	.
		0.25	0.4051	0.0021	0.6028	0.0012	1.1902	967.8	0.0116	.
		0.5	0.4061	0.0019	0.6038	0.0012	1.1847	974.7	0.0115	.
		0.75	0.4115	0.0022	0.6018	0.0011	1.1883	982.1	0.0115	.
		1	0.4097	0.0024	0.6010	0.0013	1.1888	983.5	0.0114	.
	Lasso+CTPR	0	0.4058	0.0019	0.6063	0.0013	1.1836	982.1	0.0115	0.0000
		0.25	0.4051	0.0021	0.6028	0.0012	1.1902	967.8	0.0116	0.0000
		0.5	0.4061	0.0019	0.6038	0.0012	1.1853	976.4	0.0115	0.0012
		0.75	0.4137	0.0022	0.6025	0.0011	1.2173	1035.7	0.0112	0.0606
		1	0.4194	0.0023	0.6001	0.0013	1.2720	1066.3	0.0113	0.2499
	MCP	0	0.4099	0.0019	0.5927	0.0012	0.9771	478.9	0.0164	.
		0.25	0.4088	0.0022	0.5890	0.0013	0.9788	476.3	0.0164	.
		0.5	0.4099	0.0020	0.5903	0.0012	0.9786	472.5	0.0165	.
		0.75	0.4155	0.0023	0.5876	0.0011	0.9812	475.4	0.0165	.
		1	0.4131	0.0024	0.5874	0.0012	0.9829	471.9	0.0164	.
	MCP+CTPR	0	0.4116	0.0019	0.5911	0.0012	1.0348	548.3	0.0154	0.0611
		0.25	0.4119	0.0022	0.5861	0.0012	1.0304	549.9	0.0153	0.0673
		0.5	0.4155	0.0020	0.5854	0.0011	1.0477	615.4	0.0146	0.1314
		0.75	0.4242	0.0023	0.5792	0.0010	1.0404	660.6	0.0142	0.1857
		1	0.4283	0.0024	0.5723	0.0012	1.0244	784.3	0.0131	0.3905
0.9	Lasso	0	0.4054	0.0020	0.6016	0.0012	1.1859	977.0	0.0115	.
		0.25	0.4071	0.0026	0.6015	0.0013	1.1849	973.7	0.0115	.
		0.5	0.4085	0.0021	0.6028	0.0012	1.1885	974.5	0.0115	.
		0.75	0.4073	0.0020	0.6039	0.0013	1.1869	975.0	0.0115	.
		1	0.4074	0.0023	0.6059	0.0013	1.1877	980.1	0.0115	.
	Lasso+CTPR	0	0.4054	0.0020	0.6016	0.0012	1.1859	977.0	0.0115	0.0000
		0.25	0.4071	0.0026	0.6015	0.0013	1.1849	973.7	0.0115	0.0000
		0.5	0.4089	0.0021	0.6032	0.0012	1.1966	995.5	0.0114	0.0171
		0.75	0.4139	0.0020	0.6036	0.0014	1.2483	1060.2	0.0112	0.1584
		1	0.4314	0.0021	0.5957	0.0013	1.3088	1046.8	0.0120	1.4468
	MCP	0	0.4083	0.0022	0.5891	0.0012	0.9778	476.0	0.0165	.
		0.25	0.4102	0.0027	0.5888	0.0013	0.9772	473.0	0.0165	.
		0.5	0.4121	0.0022	0.5891	0.0012	0.9787	474.0	0.0164	.
		0.75	0.4115	0.0021	0.5897	0.0013	0.9795	474.5	0.0164	.
		1	0.4109	0.0024	0.5923	0.0013	0.9798	472.4	0.0165	.
	MCP+CTPR	0	0.4105	0.0021	0.5871	0.0012	1.0338	552.0	0.0153	0.0611
		0.25	0.4140	0.0027	0.5854	0.0013	1.0372	576.1	0.0151	0.0900
		0.5	0.4192	0.0022	0.5825	0.0011	1.0408	618.8	0.0145	0.1387
		0.75	0.4239	0.0021	0.5775	0.0012	1.0312	725.5	0.0136	0.2788
		1	0.4410	0.0022	0.5619	0.0012	0.9938	1016.4	0.0118	1.6281

Abbreviation: %S=proportion of shared causal SNPs between two traits

Supplementary Table 4. Prediction R^2 and mean squared error (MSE) with h^2= (0.5,0.25), (0.5,0.75), (0.25,0.25) or (0.75,0.75) and 2 traits, 14,800 training samples, 5,000 SNPs, 500 Causal SNPs and 100 replicates.

h^2	Method	ρ_{12}	R^2	SE(R^2)	MSE	SE(MSE)	Slope	# Nz β	λ_1	λ_2
(0.5,0.25)	Lasso	0	0.4094	0.0021	0.6031	0.0012	1.1867	977.61	0.0115	.
		0.25	0.4068	0.0021	0.6011	0.0013	1.1889	971.53	0.0116	.
		0.5	0.4046	0.0021	0.6031	0.0012	1.1819	980.07	0.0115	.
		0.75	0.4068	0.0019	0.6036	0.0013	1.1793	991.64	0.0114	.
		1	0.4080	0.0022	0.6033	0.0014	1.1801	981.88	0.0115	.
	Lasso+CTPR	0	0.4094	0.0021	0.6031	0.0012	1.1867	977.61	0.0115	0.0000
		0.25	0.4068	0.0021	0.6011	0.0013	1.1889	971.53	0.0116	0.0000
		0.5	0.4046	0.0021	0.6032	0.0012	1.1820	981.49	0.0115	0.0006
		0.75	0.4080	0.0019	0.6043	0.0013	1.1982	1016.32	0.0113	0.0323
		1	0.4190	0.0022	0.6054	0.0014	1.3000	1073.61	0.0113	0.3260
	MCP	0	0.4133	0.0022	0.5891	0.0013	0.9804	475.76	0.0165	.
		0.25	0.4104	0.0022	0.5875	0.0013	0.9794	466.98	0.0165	.
		0.5	0.4085	0.0021	0.5901	0.0012	0.9753	475.13	0.0164	.
		0.75	0.4108	0.0020	0.5905	0.0014	0.9753	478	0.0164	.
		1	0.4114	0.0023	0.5907	0.0014	0.9759	472.53	0.0165	.
	MCP+CTPR	0	0.4152	0.0022	0.5875	0.0012	1.0304	548.53	0.0154	0.0596
		0.25	0.4140	0.0021	0.5845	0.0013	1.0431	571.98	0.0151	0.0923
		0.5	0.4153	0.0021	0.5840	0.0012	1.0533	658.92	0.0141	0.1628
		0.75	0.4226	0.0019	0.5800	0.0013	1.0677	829.35	0.0127	0.3678
		1	0.4367	0.0022	0.5678	0.0013	1.0851	1031.09	0.0117	1.4605
(0.5,0.75)	Lasso	0	0.4084	0.0024	0.6029	0.0011	1.1920	959.22	0.0116	.
		0.25	0.4074	0.0021	0.6028	0.0012	1.1872	976.87	0.0115	.
		0.5	0.4084	0.0023	0.6021	0.0012	1.1846	976.88	0.0115	.
		0.75	0.4054	0.0021	0.6032	0.0014	1.1838	975.65	0.0115	.
		1	0.4096	0.0022	0.6054	0.0011	1.1888	985.85	0.0115	.
	Lasso+CTPR	0	0.4084	0.0024	0.6029	0.0011	1.1920	959.22	0.0116	0.0000
		0.25	0.4075	0.0021	0.6029	0.0012	1.1885	977.49	0.0115	0.0018
		0.5	0.4132	0.0023	0.6015	0.0012	1.2275	1051.84	0.0112	0.1217
		0.75	0.4209	0.0020	0.5924	0.0013	1.2284	1054.32	0.0116	0.5741
		1	0.4644	0.0020	0.5498	0.0011	1.1735	1005.82	0.0124	16.2883
	MCP	0	0.4117	0.0024	0.5891	0.0011	0.9827	465.99	0.0166	.
		0.25	0.4108	0.0021	0.5895	0.0011	0.9793	470.73	0.0165	.
		0.5	0.4117	0.0025	0.5890	0.0012	0.9792	469.35	0.0165	.
		0.75	0.4089	0.0021	0.5902	0.0013	0.9763	476.18	0.0164	.
		1	0.4138	0.0023	0.5908	0.0011	0.9813	480.17	0.0164	.
	MCP+CTPR	0	0.4124	0.0024	0.5886	0.0011	1.0147	513.66	0.0158	0.0415
		0.25	0.4143	0.0021	0.5860	0.0011	1.0214	551.63	0.0153	0.0830
		0.5	0.4209	0.0024	0.5797	0.0011	1.0127	625.68	0.0146	0.1754
		0.75	0.4288	0.0019	0.5706	0.0012	0.9665	780.39	0.0132	0.4323

		1	0.4744	0.0020	0.5343	0.0010	0.9098	1006.67	0.0120	14.1491
(0.25,0.25)	Lasso	0	0.1422	0.0017	0.8667	0.0016	1.2201	623.94	0.0169	.
		0.25	0.1398	0.0015	0.8673	0.0018	1.2330	607.62	0.0171	.
		0.5	0.1370	0.0016	0.8659	0.0019	1.2173	613.41	0.0170	.
		0.75	0.1410	0.0014	0.8684	0.0016	1.2347	614.65	0.0171	.
		1	0.1391	0.0017	0.8666	0.0017	1.2184	617.29	0.0170	.
	Lasso+CTPR	0	0.1422	0.0017	0.8669	0.0016	1.2234	634.05	0.0168	0.0105
		0.25	0.1416	0.0014	0.8674	0.0018	1.2857	834.44	0.0151	0.2426
		0.5	0.1476	0.0015	0.8610	0.0019	1.3530	1020.46	0.0139	0.7403
		0.75	0.1656	0.0014	0.8532	0.0014	1.4217	1027.74	0.0142	2.0555
		1	0.1845	0.0016	0.8333	0.0016	1.4329	1011.76	0.0146	10.7929
	MCP	0	0.1354	0.0017	0.8692	0.0016	1.0181	296.6	0.0224	.
		0.25	0.1324	0.0016	0.8699	0.0019	1.0267	290.16	0.0225	.
		0.5	0.1298	0.0016	0.8690	0.0019	1.0184	291.45	0.0225	.
		0.75	0.1341	0.0015	0.8705	0.0016	1.0325	292.14	0.0225	.
		1	0.1321	0.0018	0.8693	0.0018	1.0228	288.63	0.0225	.
	MCP+CTPR	0	0.1404	0.0017	0.8656	0.0016	1.1180	488.08	0.0188	0.2305
		0.25	0.1439	0.0015	0.8599	0.0017	1.1170	622.25	0.0171	0.4170
		0.5	0.1486	0.0015	0.8503	0.0019	1.0430	795.43	0.0155	0.8040
		0.75	0.1679	0.0015	0.8363	0.0014	1.0063	978.12	0.0144	2.3758
		1	0.1883	0.0016	0.8128	0.0015	0.9945	1003.44	0.0144	15.6816
(0.75,0.75)	Lasso	0	0.7010	0.0017	0.3113	0.0007	1.1503	1059.97	0.0081	.
		0.25	0.6998	0.0012	0.3129	0.0007	1.1560	1060.04	0.0081	.
		0.5	0.7008	0.0016	0.3126	0.0007	1.1534	1060.48	0.0081	.
		0.75	0.7028	0.0015	0.3114	0.0006	1.1537	1060.73	0.0081	.
		1	0.6976	0.0016	0.3120	0.0008	1.1540	1061.39	0.0081	.
	Lasso+CTPR	0	0.7010	0.0017	0.3113	0.0007	1.1503	1059.97	0.0081	0.0000
		0.25	0.6998	0.0012	0.3129	0.0007	1.1560	1060.04	0.0081	0.0000
		0.5	0.7008	0.0016	0.3126	0.0007	1.1534	1060.48	0.0081	0.0000
		0.75	0.7047	0.0014	0.3119	0.0007	1.1690	1070.13	0.0081	0.0439
		1	0.7187	0.0015	0.2986	0.0007	1.2004	1031.67	0.0091	8.1278
	MCP	0	0.7099	0.0017	0.2905	0.0006	0.9856	548.08	0.0116	.
		0.25	0.7086	0.0013	0.2913	0.0007	0.9904	541.48	0.0116	.
		0.5	0.7099	0.0016	0.2912	0.0006	0.9871	550.16	0.0115	.
		0.75	0.7119	0.0014	0.2898	0.0006	0.9881	549.3	0.0116	.
		1	0.7067	0.0016	0.2908	0.0006	0.9867	551.42	0.0115	.
	MCP+CTPR	0	0.7099	0.0017	0.2905	0.0006	0.9856	548.08	0.0116	0.0000
		0.25	0.7085	0.0013	0.2916	0.0007	1.0016	556.67	0.0115	0.0171
		0.5	0.7109	0.0016	0.2902	0.0006	1.0148	594.6	0.0111	0.0593
		0.75	0.7156	0.0014	0.2862	0.0005	1.0159	636.86	0.0107	0.1322
		1	0.7296	0.0015	0.2679	0.0005	0.9978	1011.54	0.0087	14.6516

Supplementary Table 5. Prediction R^2 and mean squared error (MSE) with ρ_{ij}= 0.1, 0.5, 0.7 or 0.9 ($i \neq j$ $i \neq 1, j \neq 1$), ρ_{1i} = ρ_{i1}= 0.5, 5,000 SNPs, 500 causal SNPs, and, 100 replicates and 2-7 traits.

ρ_{ij}	# Traits	Method	R^2	SE(R^2)	MSE	SE(MSE)	Slope	# Nz β	λ_1	λ_2
0.1	2	Lasso	0.1782	0.0025	0.8253	0.0027	1.1712	414.32	0.0306	.
		Lasso+CTPR	0.2203	0.0024	0.7888	0.0023	1.2645	1122.45	0.0194	1.2979
		MCP	0.1475	0.0026	0.8529	0.0029	1.0503	146.22	0.0452	.
		MCP+CTPR	0.2158	0.0026	0.7840	0.0024	1.0018	677.47	0.0254	1.4357
	3	Lasso	0.1800	0.0023	0.8287	0.0025	1.1603	426.46	0.0301	.
		Lasso+CTPR	0.2344	0.0020	0.7912	0.0021	1.4238	1128.37	0.0193	0.5149
		MCP	0.1485	0.0024	0.8575	0.0029	1.0423	147.68	0.0451	.
		MCP+CTPR	0.2551	0.0021	0.7498	0.0021	1.0337	1111.64	0.0185	0.6822
	4	Lasso	0.1838	0.0024	0.8238	0.0028	1.1560	433.99	0.0299	.
		Lasso+CTPR	0.2379	0.0022	0.7889	0.0024	1.4512	1134.17	0.0189	0.2979
		MCP	0.1536	0.0027	0.8512	0.0033	1.0318	153.17	0.0445	.
		MCP+CTPR	0.2739	0.0023	0.7328	0.0021	1.1061	1148.66	0.0173	0.3557
	5	Lasso	0.1793	0.0023	0.8227	0.0023	1.1769	416.08	0.0304	.
		Lasso+CTPR	0.2332	0.0020	0.7895	0.0021	1.4858	1132.49	0.0190	0.2206
		MCP	0.1472	0.0024	0.8516	0.0026	1.0690	142.42	0.0454	.
		MCP+CTPR	0.2761	0.0022	0.7272	0.0020	1.1622	1160.75	0.0170	0.2516
	6	Lasso	0.1827	0.0020	0.8256	0.0027	1.1614	433.79	0.0296	.
		Lasso+CTPR	0.2347	0.0020	0.7917	0.0024	1.4417	1135.8	0.0185	0.1550
		MCP	0.1510	0.0024	0.8546	0.0032	1.0468	151.54	0.0446	.
		MCP+CTPR	0.2818	0.0022	0.7274	0.0020	1.1320	1179.28	0.0159	0.1733
	7	Lasso	0.1822	0.0024	0.8276	0.0025	1.1738	423.6	0.0303	.
		Lasso+CTPR	0.2370	0.0020	0.7934	0.0022	1.4813	1129.65	0.0189	0.1345
		MCP	0.1489	0.0025	0.8581	0.0028	1.0592	145.75	0.0455	.
		MCP+CTPR	0.2858	0.0022	0.7241	0.0017	1.1592	1169.61	0.0162	0.1392
0.5	2	Lasso	0.1813	0.0024	0.8248	0.0028	1.1608	428.74	0.0300	.
		Lasso+CTPR	0.2245	0.0020	0.7885	0.0022	1.2783	1114.28	0.0195	1.2849
		MCP	0.1500	0.0026	0.8535	0.0029	1.0420	149.52	0.0450	.
		MCP+CTPR	0.2209	0.0022	0.7822	0.0022	1.0071	672.39	0.0254	1.1759
	3	Lasso	0.1830	0.0021	0.8278	0.0028	1.1977	412.88	0.0307	.
		Lasso+CTPR	0.2363	0.0018	0.7902	0.0024	1.4284	1127.27	0.0196	0.5745
		MCP	0.1525	0.0023	0.8549	0.0032	1.0808	146.39	0.0451	.
		MCP+CTPR	0.2476	0.0021	0.7578	0.0021	0.9990	1069.8	0.0192	0.6893
	4	Lasso	0.1826	0.0026	0.8233	0.0024	1.1485	442.14	0.0297	.
		Lasso+CTPR	0.2382	0.0023	0.7848	0.0020	1.4183	1129.48	0.0192	0.3174
		MCP	0.1512	0.0028	0.8524	0.0028	1.0451	153.75	0.0449	.
		MCP+CTPR	0.2615	0.0025	0.7411	0.0017	1.0252	1133.33	0.0180	0.3905
	5	Lasso	0.1807	0.0023	0.8274	0.0027	1.1646	426.18	0.0302	.
		Lasso+CTPR	0.2389	0.0020	0.7893	0.0022	1.4694	1124.87	0.0194	0.2440
		MCP	0.1507	0.0025	0.8550	0.0032	1.0517	150.64	0.0449	.
		MCP+CTPR	0.2656	0.0022	0.7391	0.0020	1.0546	1142.12	0.0177	0.2688
	6	Lasso	0.1780	0.0023	0.8242	0.0025	1.1878	412.33	0.0307	.
		Lasso+CTPR	0.2324	0.0023	0.7890	0.0023	1.4697	1127.47	0.0193	0.1884
		MCP	0.1460	0.0027	0.8524	0.0030	1.0706	142.77	0.0454	.
		MCP+CTPR	0.2597	0.0024	0.7404	0.0021	1.0920	1139.85	0.0179	0.2223
	7	Lasso	0.1827	0.0022	0.8275	0.0026	1.1849	423.66	0.0303	.
		Lasso+CTPR	0.2399	0.0020	0.7910	0.0023	1.4892	1124.5	0.0193	0.1565

		MCP	0.1499	0.0022	0.8570	0.0030	1.0764	144.62	0.0454	.
		MCP+CTPR	0.2702	0.0022	0.7355	0.0020	1.0545	1153.38	0.0168	0.1537
0.7	2	Lasso	0.1812	0.0026	0.8256	0.0029	1.1706	421.21	0.0303	.
		Lasso+CTPR	0.2245	0.0024	0.7884	0.0024	1.2742	1118.59	0.0195	1.3595
		MCP	0.1495	0.0026	0.8544	0.0034	1.0513	145.83	0.0453	.
		MCP+CTPR	0.2201	0.0025	0.7830	0.0024	1.0011	677.73	0.0253	1.2386
	3	Lasso	0.1821	0.0025	0.8266	0.0025	1.1806	422.93	0.0303	.
		Lasso+CTPR	0.2346	0.0021	0.7894	0.0022	1.4169	1113.13	0.0199	0.6074
		MCP	0.1517	0.0027	0.8536	0.0027	1.0691	147.19	0.0452	.
		MCP+CTPR	0.2420	0.0022	0.7622	0.0021	0.9801	1032.51	0.0199	0.6968
	4	Lasso	0.1813	0.0023	0.8248	0.0027	1.1659	429.53	0.0300	.
		Lasso+CTPR	0.2365	0.0020	0.7855	0.0023	1.4101	1132.89	0.0193	0.3398
		MCP	0.1517	0.0025	0.8516	0.0031	1.0611	147.94	0.0450	.
		MCP+CTPR	0.2512	0.0022	0.7513	0.0020	0.9836	1110.69	0.0184	0.3925
	5	Lasso	0.1800	0.0027	0.8245	0.0027	1.1652	432.27	0.0299	.
		Lasso+CTPR	0.2357	0.0024	0.7858	0.0022	1.4263	1125.03	0.0192	0.2387
		MCP	0.1504	0.0028	0.8513	0.0030	1.0627	149.89	0.0448	.
		MCP+CTPR	0.2541	0.0027	0.7465	0.0020	0.9989	1124.65	0.0179	0.2702
	6	Lasso	0.1772	0.0029	0.8239	0.0028	1.1754	416.79	0.0305	.
		Lasso+CTPR	0.2352	0.0025	0.7845	0.0025	1.4506	1119.91	0.0195	0.2001
		MCP	0.1459	0.0029	0.8520	0.0033	1.0625	146.38	0.0452	.
		MCP+CTPR	0.2530	0.0027	0.7449	0.0021	1.0183	1122.78	0.0181	0.2202
	7	Lasso	0.1784	0.0024	0.8259	0.0026	1.1671	423.78	0.0303	.
		Lasso+CTPR	0.2351	0.0021	0.7872	0.0022	1.4436	1124.07	0.0194	0.1583
		MCP	0.1475	0.0026	0.8540	0.0030	1.0534	148.5	0.0451	.
		MCP+CTPR	0.2553	0.0024	0.7455	0.0019	1.0022	1140.85	0.0175	0.1671
0.9	2	Lasso	0.1799	0.0025	0.8257	0.0025	1.1798	419.93	0.0305	.
		Lasso+CTPR	0.2210	0.0023	0.7907	0.0021	1.2821	1122.21	0.0197	1.3852
		MCP	0.1496	0.0028	0.8529	0.0030	1.0771	144.4	0.0455	.
		MCP+CTPR	0.2154	0.0024	0.7859	0.0021	1.0001	690.86	0.0255	1.3393
	3	Lasso	0.1800	0.0024	0.8267	0.0027	1.1682	422.21	0.0303	.
		Lasso+CTPR	0.2322	0.0020	0.7868	0.0025	1.3618	1126.88	0.0195	0.5617
		MCP	0.1477	0.0025	0.8563	0.0030	1.0554	146.89	0.0451	.
		MCP+CTPR	0.2386	0.0022	0.7645	0.0024	0.9618	975.14	0.0205	0.6178
	4	Lasso	0.1787	0.0026	0.8234	0.0029	1.1484	437.77	0.0298	.
		Lasso+CTPR	0.2312	0.0023	0.7849	0.0027	1.3753	1127.32	0.0194	0.3387
		MCP	0.1464	0.0027	0.8536	0.0034	1.0455	148.23	0.0452	.
		MCP+CTPR	0.2392	0.0026	0.7606	0.0024	0.9559	1059.43	0.0193	0.3833
	5	Lasso	0.1830	0.0024	0.8250	0.0026	1.1413	447.35	0.0293	.
		Lasso+CTPR	0.2325	0.0020	0.7885	0.0022	1.3626	1128.69	0.0192	0.2335
		MCP	0.1519	0.0026	0.8539	0.0029	1.0519	151.09	0.0449	.
		MCP+CTPR	0.2440	0.0022	0.7617	0.0020	0.9490	1062.07	0.0187	0.2451
	6	Lasso	0.1866	0.0022	0.8255	0.0027	1.1708	442.24	0.0297	.
		Lasso+CTPR	0.2378	0.0019	0.7880	0.0023	1.3930	1127.75	0.0195	0.2001
		MCP	0.1548	0.0024	0.8544	0.0029	1.0704	148.9	0.0451	.
		MCP+CTPR	0.2472	0.0023	0.7604	0.0021	0.9621	1056.2	0.0191	0.2084
	7	Lasso	0.1793	0.0028	0.8266	0.0027	1.1671	422.69	0.0303	.
		Lasso+CTPR	0.2344	0.0025	0.7868	0.0025	1.4033	1121.52	0.0195	0.1624
		MCP	0.1485	0.0027	0.8550	0.0032	1.0510	148.33	0.0450	.

MCP+CTPR	0.2436	0.0028	0.7592	0.0023	0.9658	1057.94	0.0189	0.1661

Supplementary Table 6. Prediction R^2 and mean squared error (MSE) using summary statistics computed with 7,400, 14,800 samples or true SNPs effects with 2 traits, 14,800 training samples, 5,000 SNPs and 500 causal SNPs.

N (SS)	Method	ρ_{12}	R^2	SE(R^2)	MSE	SE(MSE)	Slope	# Nz β	λ_1	λ_2
7400	Lasso	0	0.4069	0.0021	0.6010	0.0013	1.1833	977.16	0.0115	.
		0.25	0.4055	0.0023	0.6029	0.0011	1.1836	972.74	0.0115	.
		0.5	0.4097	0.0020	0.6036	0.0014	1.1806	979.81	0.0114	.
		0.75	0.4045	0.0022	0.6022	0.0011	1.1833	987.78	0.0115	.
		1	0.4097	0.0019	0.6005	0.0012	1.1872	967.21	0.0116	.
	Lasso+CTPR	0	0.4069	0.0021	0.6010	0.0013	1.1833	977.16	0.0115	0.0000
		0.25	0.4055	0.0023	0.6029	0.0011	1.1841	974.42	0.0115	0.0012
		0.5	0.4131	0.0020	0.6029	0.0015	1.2083	1031.03	0.0113	0.0786
		0.75	0.4168	0.0021	0.5925	0.0011	1.2071	1057.53	0.0120	0.2357
		1	0.4430	0.0018	0.5657	0.0011	1.1632	1065.02	0.0153	0.6058
	MCP	0	0.4110	0.0022	0.5874	0.0013	0.9767	476.83	0.0164	.
		0.25	0.4095	0.0024	0.5895	0.0011	0.9753	478.21	0.0164	.
		0.5	0.4140	0.0021	0.5899	0.0014	0.9759	472.47	0.0164	.
		0.75	0.4086	0.0022	0.5886	0.0011	0.9760	481.61	0.0164	.
		1	0.4132	0.0020	0.5870	0.0011	0.9779	473.32	0.0164	.
	MCP+CTPR	0	0.4109	0.0022	0.5875	0.0013	1.0098	519.79	0.0158	0.0385
		0.25	0.4122	0.0024	0.5866	0.0011	1.0170	538.53	0.0156	0.0627
		0.5	0.4205	0.0021	0.5833	0.0014	1.0226	588.27	0.0152	0.1283
		0.75	0.4233	0.0021	0.5739	0.0010	1.0164	639.02	0.0155	0.2374
		1	0.4472	0.0018	0.5528	0.0011	1.0065	728.85	0.0183	0.5912
14800	Lasso	0	0.4069	0.0021	0.6010	0.0013	1.1833	977.16	0.0115	.
		0.25	0.4055	0.0023	0.6029	0.0011	1.1836	972.74	0.0115	.
		0.5	0.4097	0.0020	0.6036	0.0014	1.1806	979.81	0.0114	.
		0.75	0.4045	0.0022	0.6022	0.0011	1.1833	987.78	0.0115	.
		1	0.4097	0.0019	0.6005	0.0012	1.1872	967.21	0.0116	.
	Lasso+CTPR	0	0.4069	0.0021	0.6010	0.0013	1.1833	977.16	0.0115	0.0000
		0.25	0.4055	0.0023	0.6029	0.0011	1.1836	972.74	0.0115	0.0000
		0.5	0.4129	0.0020	0.6039	0.0015	1.2157	1042.67	0.0113	0.0933
		0.75	0.4171	0.0021	0.5939	0.0012	1.2246	1052.22	0.0124	0.3261
		1	0.4508	0.0017	0.5582	0.0011	1.1637	1120.74	0.0211	1.4311
	MCP	0	0.4110	0.0022	0.5874	0.0013	0.9767	476.83	0.0164	.
		0.25	0.4095	0.0024	0.5895	0.0011	0.9753	478.21	0.0164	.
		0.5	0.4140	0.0021	0.5899	0.0014	0.9759	472.47	0.0164	.
		0.75	0.4086	0.0022	0.5886	0.0011	0.9760	481.61	0.0164	.
		1	0.4132	0.0020	0.5870	0.0011	0.9779	473.32	0.0164	.
	MCP+CTPR	0	0.4115	0.0022	0.5869	0.0013	1.0156	527.95	0.0157	0.0446
		0.25	0.4124	0.0024	0.5865	0.0011	1.0220	544.09	0.0155	0.0689
		0.5	0.4217	0.0021	0.5824	0.0014	1.0330	596.67	0.0150	0.1426

		0.75	0.4257	0.0022	0.5718	0.0010	1.0292	676.5	0.0150	0.2974
		1	0.4579	0.0018	0.5425	0.0010	1.0314	847.53	0.0196	1.0414
True	Lasso	0	0.4069	0.0021	0.6010	0.0013	1.1833	977.16	0.0115	.
		0.25	0.4055	0.0023	0.6029	0.0011	1.1836	972.74	0.0115	.
		0.5	0.4097	0.0020	0.6036	0.0014	1.1806	979.81	0.0114	.
		0.75	0.4045	0.0022	0.6022	0.0011	1.1833	987.78	0.0115	.
		1	0.4097	0.0019	0.6005	0.0012	1.1872	967.21	0.0116	.
	Lasso+CTPR	0	0.4069	0.0021	0.6010	0.0013	1.1833	977.16	0.0115	0.0000
		0.25	0.4056	0.0023	0.6029	0.0011	1.1845	976.84	0.0115	0.0024
		0.5	0.4158	0.0020	0.6022	0.0014	1.2274	1044.35	0.0113	0.1386
		0.75	0.4253	0.0021	0.5832	0.0011	1.1961	1032.38	0.0119	0.5277
		1	0.5023	0.0018	0.4978	0.0010	1.0145	882.68	0.0144	24.1400
	MCP	0	0.4110	0.0022	0.5874	0.0013	0.9767	476.83	0.0164	.
		0.25	0.4095	0.0024	0.5895	0.0011	0.9753	478.21	0.0164	.
		0.5	0.4140	0.0021	0.5899	0.0014	0.9759	472.47	0.0164	.
		0.75	0.4086	0.0022	0.5886	0.0011	0.9760	481.61	0.0164	.
		1	0.4132	0.0020	0.5870	0.0011	0.9779	473.32	0.0164	.
	MCP+CTPR	0	0.4115	0.0022	0.5870	0.0013	1.0194	531.48	0.0156	0.0489
		0.25	0.4129	0.0024	0.5860	0.0011	1.0241	553.04	0.0154	0.0759
		0.5	0.4234	0.0021	0.5807	0.0014	1.0341	606.61	0.0148	0.1650
		0.75	0.4321	0.0021	0.5652	0.0010	1.0252	708.19	0.0142	0.3929
		1	0.5023	0.0018	0.4977	0.0010	1.0045	818.36	0.0158	23.7300

Abbreviation: N(SS)=number of samples to generate summary statistics or true coefficients

Supplementary Table 7. Information on UK Biobank, NHS/HPFS/PHS cohort datasets.

Data	#Samples	Phenotype	Ethnicity	Genotype Platform	Reference Panel
UK Biobank	456,837	HGT,BMI	European	UK BiLEVE array	UK10K+
		HIP,WST,WHR		UK Biobank Axiom Array	1000Genomes Ph3
NHS/HPFS/PHS	20,769	HGT,BMI	European	Affymatrix 6.0	1000Genomes Ph1
				Illumina HumanHap 550,610,660	
				Immumina OmniExpress	

Supplementary Table 8. NHS/HPFS/PHS cohort data for simulation studies and real data analysis.

Data	Study	Disease	Platform	N	Total N
Affymetrix	NHS	T2D	Affymetrix 6.0	3285	8065
	HPFS	T2D	Affymetrix 6.0	2487	
	NHS	CHD	Affymetrix 6.0	1146	
	HPFS	CHD	Affymetrix 6.0	1147	
Illumina	NHS	Glaucoma	Illumina 660	804	6787
	HPFS	Glaucoma	Illumina 660	472	
	NHS	KS	Illumina 610	489	
	NHS2	KS	Illumina 610	635	
	HPFS	KS	Illumina 610	544	
	NHS	BrCa	Illumina 550	2256	
	NHS2	BrCa	Illumina 610	289	
	HPFS	AdvCap	Illumina 610	394	
	PHS	AdvCap	Illumina 610	540	
	NHS	Pancreas	Illumina 550	162	
	HPFS	Pancreas	Illumina 550	101	
	PHS	Pancreas	Illumina 550	101	
OmniExpress	NHS	GOUT	OmniExpress	700	5917
	HPFS	GOUT	OmniExpress	1415	
	NHS	Colon	OmniExpress	1165	
	HPFS	Colon	OmniExpress	458	
	PHS	Colon	OmniExpress	664	
	NHS	Endometrial	OmniExpress	726	
	NHS	MD	OmniExpress	789	
Total	12 Studies	11 Diseases	6 Platforms	20769	20769

Abbreviations: T2D=Type 2 Diabetes, CHD=Coronary Heart Disease, KS=Kidney Stone, BrCa=Breast Cancer, AdvCap=Advanced Cancer of the Prostate in Sweden, MD=Mammographic Density

Supplementary Table 9. Prediction R2 and mean squared error (MSE) computed with two different sample sizes (N=30K, 437K) using PRS, LDpred, MTAG, MTGBLUP and CTPR methods. We considered height (HGT) as a primary trait and body mass index (BMI) as a secondary trait and used NHS/HPFS/PHS cohort or UK Biobank data as a validation set separately.

N	Validation	Primary	Second	Data(2nd)	Method	R^2	MSE	Slope
30K	NHS/HPFS /PHS	HGT	x	x	PRS	0.0701	7364.63	-0.0497
					LDpred	0.0899	39.6256	5.0644
				x	STGBLUP	0.0862	36.4127	0.8483
					MCP	0.0917	36.4183	0.7679
					Lasso	0.1042	35.7461	0.8361
			BMI	UKB Stat	PRS+MTAG	0.0698	7404.57	-0.0495
					LDpred+MTAG	0.0891	39.5739	5.3252
				UKB Ind	MTGBLUP	0.0851	36.4773	0.8357
				UKB Stat	MCP+CTPR	0.1132	35.2870	1.1247
					Lasso+CTPR	0.1132	35.3078	1.1529
30K	UKB	HGT	x	x	PRS	0.0883	5225.09	-0.0574
					LDpred	0.1135	39.1111	5.8195
				x	STGBLUP	0.1142	35.3496	1.0360
					MCP	0.1142	35.4198	0.8883
					Lasso	0.1313	34.6628	0.9863
			BMI	UKB Stat	PRS+MTAG	0.0883	5242.49	-0.0572
					LDpred+MTAG	0.1129	39.18	6.1366
				UKB Ind	MTGBLUP	0.1142	35.3490	1.0263
				UKB Stat	MCP+CTPR	0.1454	34.5195	1.3680
					Lasso+CTPR	0.1458	34.5708	1.4057
437K	NHS/HPFS /PHS	HGT	x	x	PRS	0.1427	666.85	-0.1093
					LDpred	0.2218	36.3075	4.5815
				x	MCP	0.2609	60.8012	0.8041
					Lasso	0.2796	39.7619	1.0116
			BMI	UKB Stat	PRS+MTAG	0.1412	686.60	-0.1082
					LDpred+MTAG	0.2226	36.9847	4.7896
				UKB Stat	MCP+CTPR	0.2922	29.1754	1.0650
					Lasso+CTPR	0.2978	28.8956	1.0395
437K	UKB	HGT	x	x	PRS	0.2233	763.11	-0.1306
					LDpred	0.3654	35.5245	5.3223
				x	MCP	0.3776	25.0103	0.9397
					Lasso	0.3908	24.7200	1.1614
			BMI	UKB Stat	PRS+MTAG	0.2220	753.55	-0.1296
					LDpred+MTAG	0.3584	36.4958	5.7383
				UKB Stat	MCP+CTPR	0.4248	24.0173	1.0331
					Lasso+CTPR	0.4284	23.2622	1.0276

Abbreviations: UKB=UK Biobank, UKB Stat=Summary statistics in UK Biobank, UKB Ind=Individual-level data in UK Biobank

Supplementary Table 10. Prediction R^2 and mean squared error (MSE) using PRS, LDpred, MTAG, MTGBLUP and CTPR methods. We considered height (HGT) as a primary trait and hip circumference (HIP), waist circumference (WST) and waist-hip ratio (WHR) as a secondary trait and used NHS/HPFS/PHS cohort or UK Biobank data as a validation set separately.

N	Validation	Primary	Second	Data(2nd)	Method	R2	MSE	Slope
30K	NHS/HPFS /PHS	HGT	HIP	UKB Stat	PRS+MTAG	0.0704	7326.08	-0.0497
					LDpred+MTAG	0.0898	39.5744	5.3455
				UKB Ind	MTGBLUP	0.0847	36.5702	0.8011
				UKB Stat	MCP+CTPR	0.1031	35.7451	1.1985
					Lasso+CTPR	0.1046	35.7873	1.2935
			WST	UKB Stat	PRS+MTAG	0.0702	7372.483	-0.0496
					LDpred+MTAG	0.0894	39.593	5.3389
				UKB Ind	MTGBLUP	0.0863	36.4306	0.8353
				UKB Stat	MCP+CTPR	0.1048	35.8116	1.3233
					Lasso+CTPR	0.1053	35.8676	1.3843
			WHR	UKB Stat	PRS+MTAG	0.0700	7352.241	-0.0496
					LDpred+MTAG	0.0893	39.5258	5.3287
				UKB Ind	MTGBLUP	0.0855	36.4448	0.8439
				UKB Stat	MCP+CTPR	0.1052	35.7934	1.3192
					Lasso +CTPR	0.1056	35.8506	1.3794
			BMI/HIP WST/WHR	UKB Stat	PRS+MTAG	0.0687	7424.239	-0.0492
					LDpred+MTAG	0.0878	39.6229	5.2641
				UKB Ind	MTGBLUP	0.0862	36.4313	0.8354
				UKB Stat	MCP+CTPR	0.1131	35.2955	1.1325
					Lasso+CTPR	0.1132	35.3156	1.1613
30K	UKB	HGT	HIP	UKB Stat	PRS+MTAG	0.0883	5196.228	-0.0573
					LDpred+MTAG	0.1125	39.1414	6.1236
				UKB Ind	MTGBLUP	0.1094	35.5446	0.9584
				UKB Stat	MCP+CTPR	0.1307	35.1600	1.4308
					Lasso+CTPR	0.1338	35.2471	1.5567
			WST	UKB Stat	PRS+MTAG	0.0883	5233.249	-0.0573
					LDpred+MTAG	0.1128	39.1812	6.1306
				UKB Ind	MTGBLUP	0.1126	35.4110	1.0090
				UKB Stat	MCP+CTPR	0.1344	35.2894	1.5963
					Lasso+CTPR	0.1352	35.3844	1.6747
			WHR	UKB Stat	PRS+MTAG	0.0884	5186.579	-0.0573
					LDpred+MTAG	0.0983	39.5567	5.4920
				UKB Ind	MTGBLUP	0.1141	35.3553	1.0333
				UKB Stat	MCP+CTPR	0.1346	35.2726	1.5898
					Lasso+CTPR	0.1354	35.3671	1.6677
			BMI/HIP WST/WHR	UKB Stat	PRS+MTAG	0.0876	5183.02	-0.0570
					LDpred+MTAG	0.1119	39.1691	6.0686

UKB Ind	MTGBLUP	0.1140	35.3543	1.0331
UKB Stat	MCP+CTPR	0.1471	34.4871	1.3850
	Lasso+CTPR	0.1474	34.5407	1.4233

Supplementary Table 11. Prediction R^2 and mean squared error (MSE) with different sample sizes (N=30K,200K,437K) and number of traits (T=2,3,4) using PRS, LDpred, MTAG, MTGBLUP and CTPR methods when genetic correlation between two traits=0.15.

N	#Traits	ρ	Method	R^2	MSE	Slope
30K	2	0.15	PRS	0.0914	8154.01	-0.0094
			LDpred	0.1076	1.4363	0.9077
			STGBLUP	0.1043	0.8869	0.9854
			MCP	0.1145	0.8867	0.9812
			LAS	0.1235	0.8878	1.4261
			PRS+MTAG	0.0924	25428.78	-0.0088
			LDpred+MTAG	0.1089	3.0516	0.8644
			MTGBLUP	0.1052	0.8858	0.9902
			MCP+CTPR	0.1294	0.8737	1.44249
			LAS+CTPR	0.1420	0.8560	1.28018
30K	3	0.15	PRS	0.0914	8154.01	-0.0094
			LDpred	0.1076	1.4363	0.9077
			STGBLUP	0.1043	0.8869	0.9854
			MCP	0.1145	0.8867	0.9812
			LAS	0.1235	0.8878	1.4261
			PRS+MTAG	0.0954	19464.81	-0.0089
			LDpred+MTAG	0.1118	2.2218	0.8582
			MTGBLUP	0.1088	0.8943	1.0044
			MCP+CTPR	0.1344	0.8659	1.3554
			LAS+CTPR	0.1472	0.8664	1.52160
30K	4	0.15	PRS	0.0914	8154.01	-0.0094
			LDpred	0.1076	1.4363	0.9077
			STGBLUP	0.1043	0.8869	0.9854
			MCP	0.1145	0.8867	0.9812
			LAS	0.1235	0.8878	1.4261
			PRS+MTAG	0.0979	19004.94	-0.0089
			LDpred+MTAG	0.1138	2.4579	0.8563
			MTGBLUP	0.1124	0.8636	1.0074
			MCP+CTPR	0.1380	0.8727	1.6093
			LAS+CTPR	0.1507	0.8749	1.7011
200K	2	0.15	PRS	0.1559	4129.56	-0.0163
			LDpred	0.2435	2.5768	0.6544
			MCP	0.2842	0.7645	0.4763
			LAS	0.2913	0.7404	0.6059
			PRS+MTAG	0.1557	917.07	-0.0159
			LDpred+MTAG	0.2477	0.8001	1.1117
			MCP+CTPR	0.3049	0.7584	0.5494
			LAS+CTPR	0.3111	0.7396	0.6329

437K	2	0.15	PRS	0.1902	3270.83	-0.0179
			LDpred	0.3453	141.7601	0.1797
			MCP	0.3848	0.7292	0.5584
			LAS	0.3948	0.6948	0.6984
			PRS+MTAG	0.1928	633.16	-0.0173
			LDpred+MTAG	0.3476	0.8239	0.8867
			MCP+CTPR	0.4101	0.6998	0.6994
			LAS+CTPR	0.4112	0.6597	0.7991

Supplementary Table 12. Prediction accuracy using only unrelated testing samples (N=13,685) and all testing samples (N=20,000) and subsampling p-values to check how related samples between training and testing sets from UK Biobank have impact on potential inflation of prediction accuracy.

N	Validation	Primary	Second	Method	R^2 (All)	R^2 (Unrel)	P_{Diff}
30K	NHS/HPFS /PHS	HGT	x	MCP	0.0917	0.0912	0.9820
				LAS	0.1042	0.1038	0.9500
			BMI	MCP+CTPR	0.1132	0.1122	0.8980
				LAS+CTPR	0.1132	0.1123	0.9040
	UKB	HGT	x	MCP	0.1142	0.1151	0.8340
				LAS	0.1313	0.1336	0.6740
			BMI	MCP+CTPR	0.1454	0.1465	0.8760
				LAS+CTPR	0.1458	0.1467	0.8960
437K	NHS/HPFS /PHS	HGT	x	MCP	0.2609	0.2592	0.8540
				LAS	0.2796	0.2779	0.8820
			BMI	MCP+CTPR	0.2922	0.2893	0.7420
				LAS+CTPR	0.2978	0.2944	0.7020
	UKBio	HGT	x	MCP	0.3776	0.3740	0.6040
				LAS	0.3908	0.3862	0.5160
			BMI	MCP+CTPR	0.4248	0.4191	0.4220
				LAS+CTPR	0.4284	0.4234	0.4800

Abbreviations: R^2 (All): prediction accuracy using all testing samples, R^2 (Unrel): prediction accuracy using unrelated testing samples, P_{Diff}: subsampling p-values for testing mean difference between R^2 (All) and R^2 (Unrel).

References

1. Daetwyler, H.D., Villanueva, B. & Woolliams, J.A. Accuracy of predicting the genetic risk of disease using a genome-wide approach. *PLoS One* **3**, e3395 (2008).
2. Visscher, P.M., Yang, J. & Goddard, M.E. A commentary on ‘common SNPs explain a large proportion of the heritability for human height’by Yang et al.(2010). *Twin Research and Human Genetics* **13**, 517-524 (2010).
