# Supplementary material for: Efficient cross-trait penalized regression increases prediction accuracy in large cohorts using secondary phenotypes
Source: Nat Commun. 2019 Feb 4;10:569. doi: 10.1038/s41467-019-08535-0 (PMC6361917; doi:10.1038/s41467-019-08535-0)
Supplement: Supplementary file 3 — Reporting Summary [file 41467_2019_8535_MOESM3_ESM.pdf]

nature research

Corresponding author(s): Dr. Liming Liang

Last updated by author(s): Jan 10, 2019

Reporting Summary

Nature Research wishes to improve the reproducibility of the work that we publish. This form provides structure for consistency and transparency in reporting. For further information on Nature Research policies, see Authors & Referees and the Editorial Policy Checklist.

Statistics

For all statistical analyses, confirm that the following items are present in the figure legend, table legend, main text, or Methods section.

n/a	Confirmed	
☐	☒	The exact sample size (*n*) for each experimental group/condition, given as a discrete number and unit of measurement
☐	☒	A statement on whether measurements were taken from distinct samples or whether the same sample was measured repeatedly
☐	☒	The statistical test(s) used AND whether they are one- or two-sided<br>*Only common tests should be described solely by name; describe more complex techniques in the Methods section.*
☐	☒	A description of all covariates tested
☐	☒	A description of any assumptions or corrections, such as tests of normality and adjustment for multiple comparisons
☐	☒	A full description of the statistical parameters including central tendency (e.g. means) or other basic estimates (e.g. regression coefficient) AND variation (e.g. standard deviation) or associated estimates of uncertainty (e.g. confidence intervals)
☐	☒	For null hypothesis testing, the test statistic (e.g. *F*, *t*, *r*) with confidence intervals, effect sizes, degrees of freedom and *P* value noted<br>*Give P values as exact values whenever suitable.*
☐	☒	For Bayesian analysis, information on the choice of priors and Markov chain Monte Carlo settings
☐	☒	For hierarchical and complex designs, identification of the appropriate level for tests and full reporting of outcomes
☐	☒	Estimates of effect sizes (e.g. Cohen's *d*, Pearson's *r*), indicating how they were calculated

Our web collection on statistics for biologists contains articles on many of the points above.

Software and code

Policy information about availability of computer code

Data collection	No software was used to collect the data. We used UK Biobank cohort data (application number 16549) for our analyses.
Data analysis	We developed our own software, CTPR on top of "Armadillo" which is C++ library for linear algebra and scientific computing. The complete pipeline used to install and execute the CTPR, including required computing resources, input file formats and optional parameters, is provided on GitHub. To compare with our software, we used LDpred, MTGBLUP and MTAG softwares. The version and web site of all software are described in the manuscript.

For manuscripts utilizing custom algorithms or software that are central to the research but not yet described in published literature, software must be made available to editors/reviewers. We strongly encourage code deposition in a community repository (e.g. GitHub). See the Nature Research guidelines for submitting code & software for further information.

Data

Policy information about availability of data

All manuscripts must include a data availability statement. This statement should provide the following information, where applicable:

- Accession codes, unique identifiers, or web links for publicly available datasets
- A list of figures that have associated raw data
- A description of any restrictions on data availability

All Predictors for human height by aid of BMI with 437K training samples from UK Biobank using PRS, LDpred, MCP, Lasso, PRS+MTAG, LDpred+MTAG, MCP+CTPR and Lasso+CTPR are available for academic uses at http://lianglab.rc.fas.harvard.edu/CTPR/. All other data are available from the corresponding author upon reasonable request.

Field-specific reporting

Please select the one below that is the best fit for your research. If you are not sure, read the appropriate sections before making your selection.

☒ Life sciences ☐ Behavioural & social sciences ☐ Ecological, evolutionary & environmental sciences

For a reference copy of the document with all sections, see nature.com/documents/nr-reporting-summary-flat.pdf

Life sciences study design

All studies must disclose on these points even when the disclosure is negative.

Sample size	Over 500,000 subjects with their phenotypes in the UK Biobank, 488,377 subjects were genotyped. Individuals of non-European ancestry were excluded to be consistent with NHS/HPFS/PHS cohort data, which results in 456,837 samples. We used full UK Biobank samples for our analysis.
Data exclusions	No data were excluded from our analyses.
Replication	All the prediction results in the manuscript are replicable.
Randomization	For n-fold cross-validation, the data for the primary trait were randomly partitioned into n equal-sized subsets, where n-1 subsets were used as a training set and the remaining one as a validation set. Also, when part of samples were used for the analysis, we randomly selected individuals from full samples.
Blinding	Because we did not allocate any groups for our analyses, blinding is not relevant to our study.

Reporting for specific materials, systems and methods

We require information from authors about some types of materials, experimental systems and methods used in many studies. Here, indicate whether each material, system or method listed is relevant to your study. If you are not sure if a list item applies to your research, read the appropriate section before selecting a response.

Materials & experimental systems

n/a	Involved in the study	
☒	☐	Antibodies
☒	☐	Eukaryotic cell lines
☒	☐	Palaeontology
☒	☐	Animals and other organisms
☐	☒	Human research participants
☒	☐	Clinical data

Methods

n/a	Involved in the study	
☒	☐	ChIP-seq
☒	☐	Flow cytometry
☒	☐	MRI-based neuroimaging

Human research participants

Policy information about studies involving human research participants

Population characteristics	UK Biobank is a prospective cohort study of over 500,000 individuals from across the United Kingdom. Participants, aged between 40 and 69, were invited to one of 22 centres across the UK between 2006 and 2010. Blood, urine and saliva samples were collected, physical measurements were taken, and each individual answered an extensive questionnaire focused on questions of health and lifestyle.
Recruitment	UK Biobank recruited 500,000 people aged between 40-69 years in 2006-2010 from across the country to take part in the project. They have undergone measures, provided blood, urine and saliva samples for future analysis, detailed information about themselves and agreed to have their health followed. Over many years this will build into a powerful resource to help scientists discover why some people develop particular diseases and others do not.
Ethics oversight	UK Biobank is a major national and international health resource, and a registered charity in its own right, with the aim of improving the prevention, diagnosis and treatment of a wide range of serious and life-threatening illnesses – including cancer, heart diseases, stroke, diabetes, arthritis, osteoporosis, eye disorders, depression and forms of dementia.

Note that full information on the approval of the study protocol must also be provided in the manuscript.
